# Development of AB_3_-Type Novel Phthalocyanine and Porphyrin Photosensitizers Conjugated with Triphenylphosphonium for Higher Photodynamic Efficacy

**DOI:** 10.1021/acsomega.2c05814

**Published:** 2022-10-19

**Authors:** Emel Önal, Özge Tüncel, İpek Erdoğan Vatansever, Mohamad Albakour, Gizem Gümüşgöz Çelik, Tuğba Küçük, Bünyamin Akgül, Ayşe Gül Gürek, Serdar Özçelik

**Affiliations:** † Department of Chemistry, Gebze Technical University, Gebze 41400 Kocaeli, Turkey; ‡ Faculty of Engineering, Doğuş University, Ümraniye, Istanbul 34775, Turkey; § Faculty of Science, Department of Chemistry, Izmir Institute of Technology, Urla, Izmir 35430, Turkey; ∥ Faculty of Science, Department of Molecular Biology and Genetics, Izmir Institute of Technology, Urla, Izmir 35430, Turkey

## Abstract

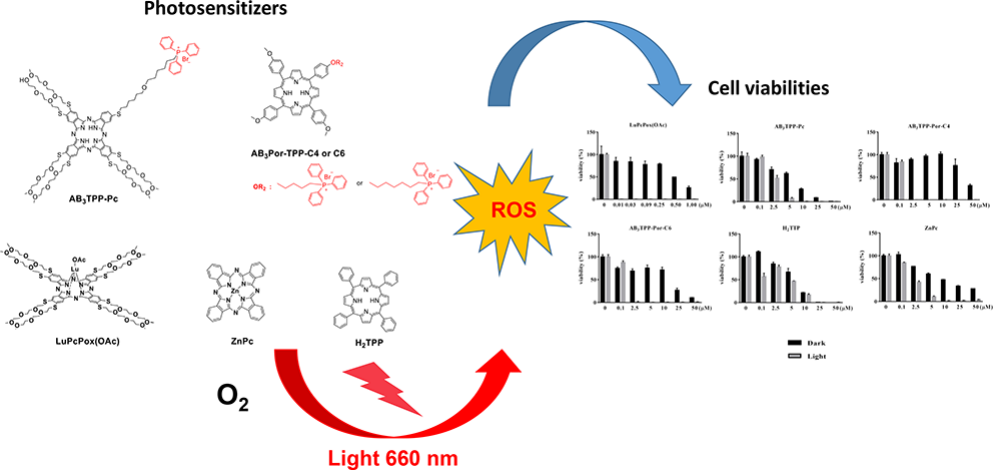

There are a number of lipophilic cations that can be chosen; the triphenylphosphonium (TPP) ion is particularly unique for mitochondrion targeting, mainly due to its simplicity in structure and ease to be linked to the target molecules. In this work, mitochondrion-targeted AB_3_-type novel phthalocyanine and porphyrin photosensitizers (PSs) were synthesized and their photophysical photochemical properties were defined. Fluorescence quantum yields (Φ_F_) are 0.009, 0.14, 0.13, and 0.13, and the singlet-oxygen quantum yields (Φ_Δ_) are 0.27, 0.75, 0.57, and 0.58 for **LuPcPox(OAc)**, **AB**_**3**_**TPP-Pc**, **AB**_**3**_**TPP-Por-C4**, and **AB**_**3**_**TPP-Por-C6**, respectively. To evaluate the photodynamic efficacy of the TPP-conjugated PS cell viabilities of A549 and BEAS-2B lung cells were comparatively measured and IC-50 values were determined. **AB**_**3**_**TPP-Por-C4**, **AB**_**3**_**TPP-Por-C6**, and **AB**_**3**_**TPP-Pc** compounds compared to the reference molecules **ZnPc** and **H**_**2**_**TPP** were found to be highly cytotoxic (sub-micromolar concentration) under the light. **LuPcPox(OAc)** is the most effective molecule regarding cell killing (the activity). The cell killing of the TPP-conjugated porphyrin derivatives exhibits a similar response compared to **LuPcPox(OAc)** when the light absorbing factor of the PS is normalized at 660 nm: TPP-conjugated porphyrins absorb less light (lower extinction coefficient) but produce more radical species (higher singlet-oxygen quantum yield) and therefore effectively kill the cells. The singlet oxygen-producing capacity of **AB**_**3**_**TPP-Pc** is almost 3 times higher compared to **LuPcPox(OAc)** and 50% more efficient with respect to ZnPc, suggesting that TPP-conjugated phthalocyanine may serve as a good photosensitizer for photodynamic therapy (PDT). The high singlet oxygen generation capacity of these novel TPP-conjugated porphyrin and phthalocyanine PS suggests that they might be useful for PDT requiring lower photosensitizer concentration and reduced energy deposited through less light exposure.

## Introduction

The mitochondrion is a small subcellular organelle generally considered as the cellular powerhouse that generates most cellular energy in the form of adenosine triphosphate.^1,2^ During the past 2 decades, research on mitochondrion-dependent cellular signaling and cell death has increased throughout the world. The current thinking is based on developing small molecules that are taken up by mitochondria and act as either probes of mitochondrial function or therapeutics in vivo to fully understand the mitochondrial function of biological processes in health and cancer-like diseases.^3^ The most well-known applicable method to target small neutral molecules to the mitochondrial matrix is by conjugation to a lipophilic cation.^4−6^ The distinctive feature of lipophilic cations is that their positive charge is delocalized over a large and hydrophobic surface area. Triphenylphosphine (TPP) cations have a strong tendency to bind to the surface of phospholipid bilayers in a potential energy well close to the membrane surface, with the “cargo” attached to the TPP cation dipping into the membrane. Increasing the hydrophobicity enhances this tendency to bind to the inner membrane, thus they can pass easily through phospholipid bilayers, enabling their accumulation into the mitochondrial matrix. Since then, the uptake of the lipophilic methyltriphenylphosphonium, tetraphenylphosphonium, and triphenylphosphonium lipophilic cations have been widely used^7−9^ Using the TPP moiety to generate mitochondrion-targeted lipophilic cations has a number of advantages as it is chemically relatively easy to introduce a TPP into a compound usually by displacing a leaving group by reaction with a TPP moiety.^10^ This advantage has led to a range of mitochondrion-targeted compounds based on TPP being widely used by a number of groups to target antioxidants and bioactive molecules.^11−13^

Compared with chemotherapy and radiotherapy, photodynamic therapy (PDT) is considered a more safe, more efficient, and minimally invasive cancer treatment.^14^ Among the cell organelles mitochondria have been reported to play a major role in photodynamic cell death.^15^ One of the limitations of PDT involves a lack of tumor selectivity of the photosensitizer, leading to the injury of normal tissue upon exposure to light. Photosensitizers (PSs) are of great importance when considering the key components of PDT. Some PSs, such as protoporphyrin^16,17^ and phthalocyanine (Pc)^15,18−21^ have been known for mitochondrion localization for a long time, and PSs that localize to mitochondria are reported to be more efficient in killing cells than those that localize at other cellular sites.^22,23^ In light of this information, the PDT efficiency of PSs can be enhanced by improving their intracellular targeting ability, thanks to TPP cations that can accumulate selectively several 100-fold within mitochondria.

**Figure 1:**
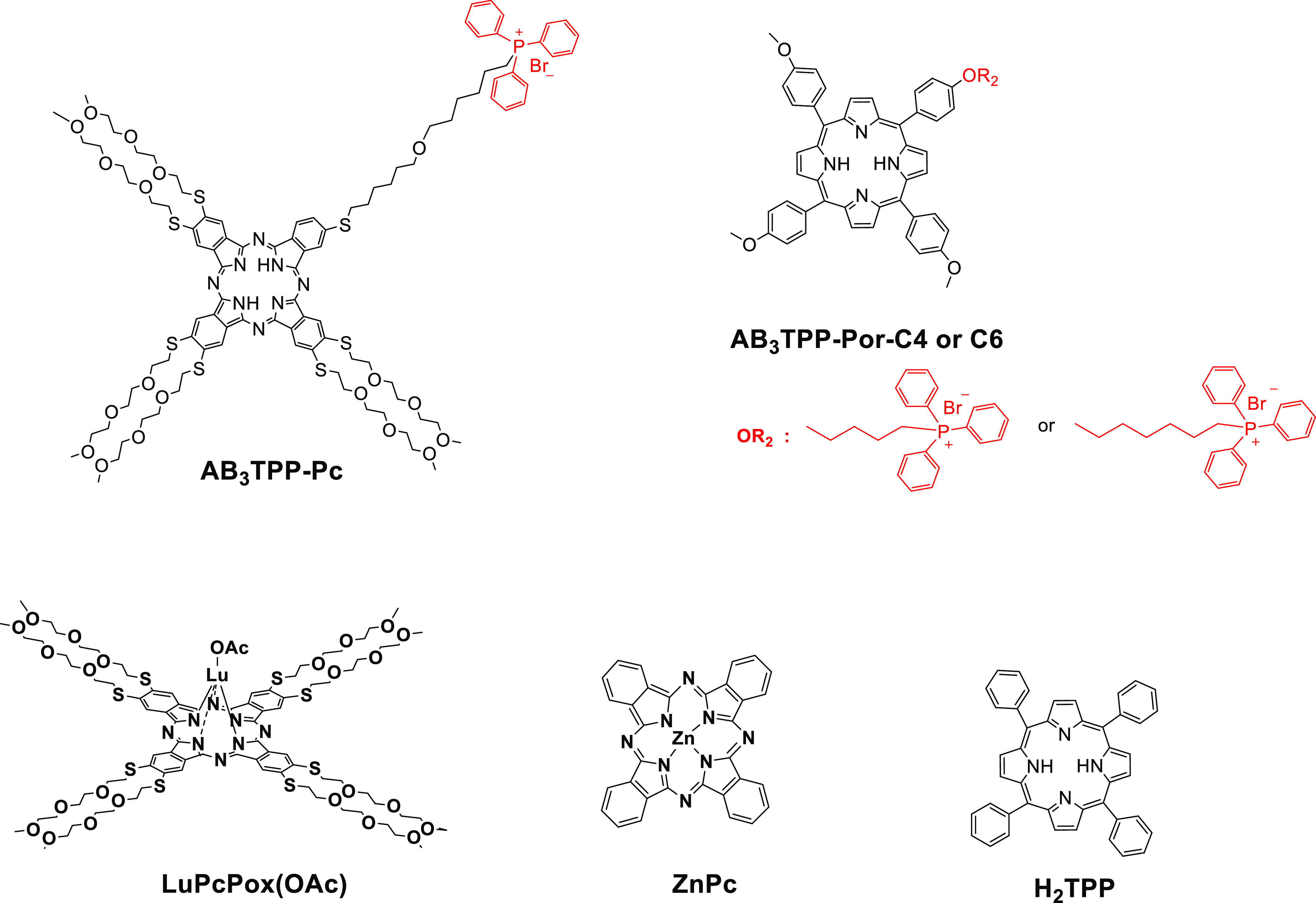
Molecular structures of phthalocyanine and porphyrin derivatives involved in this study.

In this report, the objective of our study was to present novel AB_3_-type phthalocyanine and porphyrin-based mitochondria-targeted PSs which were conjugated by TPP for the PDT application area. In our previous work^24^ which was based on the synthesis and evaluation of the photodynamic efficacy of phthalocyanine-based PS for PDT, especially the **LuPcPox(OAc)** phthalocyanine derivative having only polyoxyethylene chains (Figure 1) was determined as the best candidate photosensitizer among other phthalocyanine complexes used in that study. This complex without the TPP group induced concentration-dependent cell killing and responded to light irradiation, reducing the IC_50_ values down to the nanomolar range. Based on this important result, regardless of the metal effect, we wanted to examine the efficacy of the presence of the mitochondrion-targeted TPP group on the structure this time. Thus, we designed a new phthalocyanine compound **AB_3_TPP-Pc** derivative containing the TPP group. In addition, new porphyrin structures **AB_3_TPP-Por-C4** and **PP-Por-C6**, which are known to have high photodynamic activity as well, produced TPP group-bearing derivatives for the same purpose (Figure 1). To determine the effectiveness of TPP groups on novel PS, their photophysical/photochemical properties and the effect on cancer cell killing abilities were comparatively investigated using **ZnPc** and **H**_**2**_**TPP** as references.

## Results and Discussion

### Synthesis

**Scheme 1:**
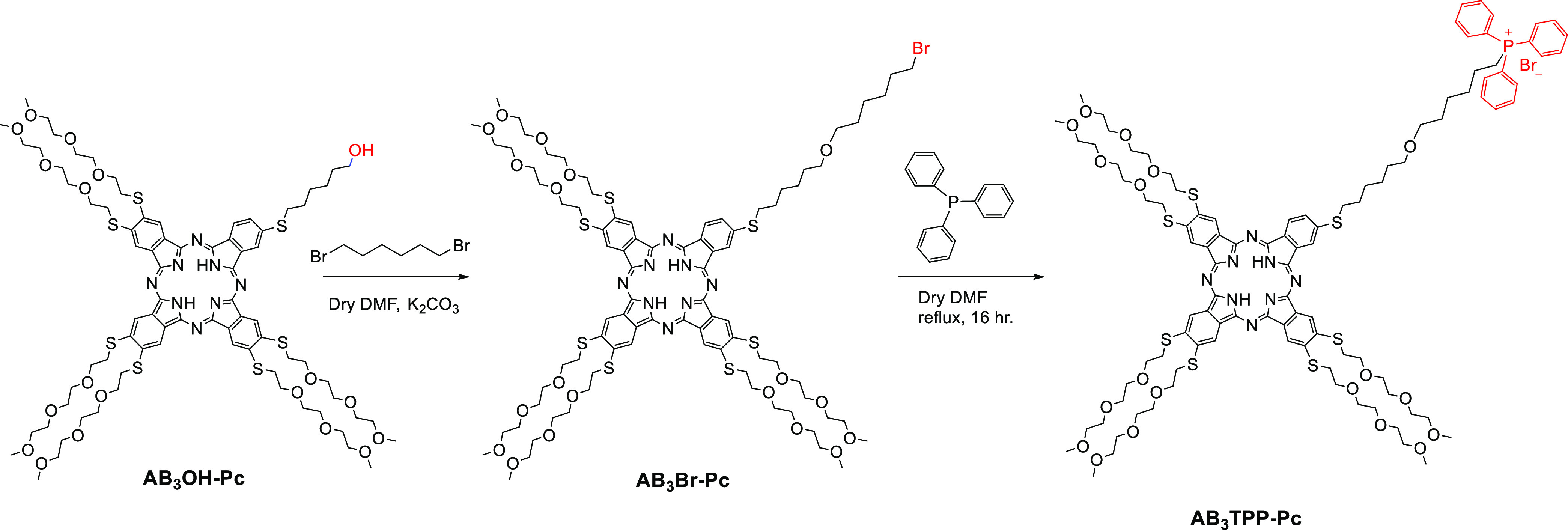
Synthesis of TPP-Conjugated Phthalocyanine **AB**_**3**_**TPP-Pc** Compound

**Scheme 2:**
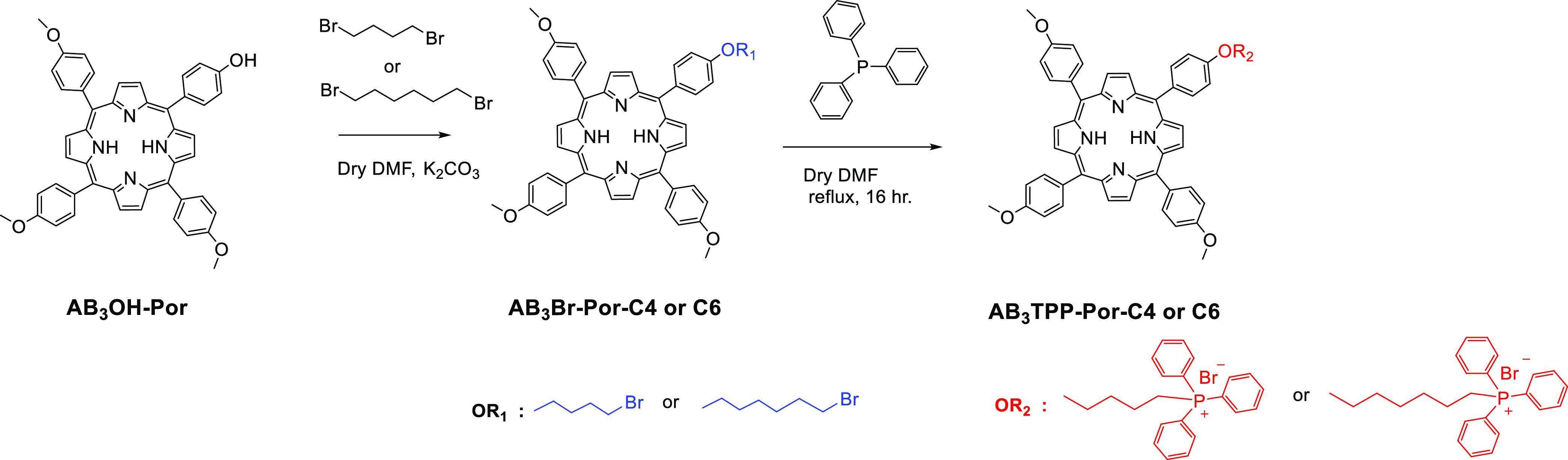
Synthesis of TPP-Conjugated Porphyrin **AB**_**3**_**TPP-Por-C4** and **AB**_**3**_**TPP-Por-C6** Compounds

An ideal photosensitizer requires properties such as high biocompatibility and efficient cellular internalization. Therefore, many polyethylene glycol-substituted Pcs have been reported^25−29^ including our previous work.^30^ Thanks to lipophilic TPP cations which have a number of advantages as explained above, TPP-conjugated porphyrin syntheses are widely covered in the literature,^31−35^ but not much mitochondrion-targeting phthalocyanine with triphenylphosphonium groups has been reported so far, to the best of our knowledge.^36−38^ In this work, the synthetic procedures for the preparation of target TPP-conjugated phthalocyanine and porphyrin PS are developed as illustrated in Schemes 1 and 2. The synthesis procedures for the precursor 5-(4-hydroxyphenyl)-10,15,20-tri(4-methoxyphenyl)porphyrin (**AB**_**3**_**Por-OH**) and its 3-bromobuthoxyloxyphenyl-substituted derivative have been published by Wang et al. who demonstrated that the fluorescence intensity and quantum yield of the porphyrin were increased by substituting it with methoxy groups.^39^ In our work, we started with the same precursor and worked with different carbon number chains as intermediate compounds 5-[4-(4-bromobutyloxy)phenyl]-10,15,20-tri(4-methoxyphenyl) porphyrin (**AB**_**3**_**Br-Por-C4**) and 5-[4-(6-bromohexyloxy)phenyl]-10,15,20-tri(4-methoxyphenyl) porphyrin (**AB**_**3**_**Br-Por-C6**) by following the same synthetic procedure. In case of phthalocyanine, the synthesis of **AB**_**3**_**OH-Pc** as the starting compound was described in our previous work.^24^ Then, excessive 1,6-dibromohexane was reacted with phthalocyanine in the presence of potassium carbonate in dimethylformamide (DMF), leading to brominated phthalocyanine (**AB**_**3**_**Br-Pc)** using the same synthesis procedure as in porphyrins. Modifications of brominated porphyrin (**AB**_**3**_**Br-Por-C4** or **AB**_**3**_**Br-Por-C6)** and phthalocyanine (**AB**_**3**_**Br-Pc)** derivatives with the mitochondrial target functional group TPP to obtain **AB**_**3**_**TPP-Por-C4**, **AB**_**3**_**TPP-Por-C6**, and **AB**_**3**_**TPP-Pc** compounds were made following a procedure applied in one of our previous reports^24^ with the reaction of excess TPP in dry DMF (Schemes 1 and 2). The structures illustrated in Schemes 1 and 2 were characterized by ^1^H NMR, MALDI-mass, NIR-IR, and UV–vis spectroscopic methods (Figures S1–S12).

### Ground-State Electronic Absorption Spectra

**Figure 2:**
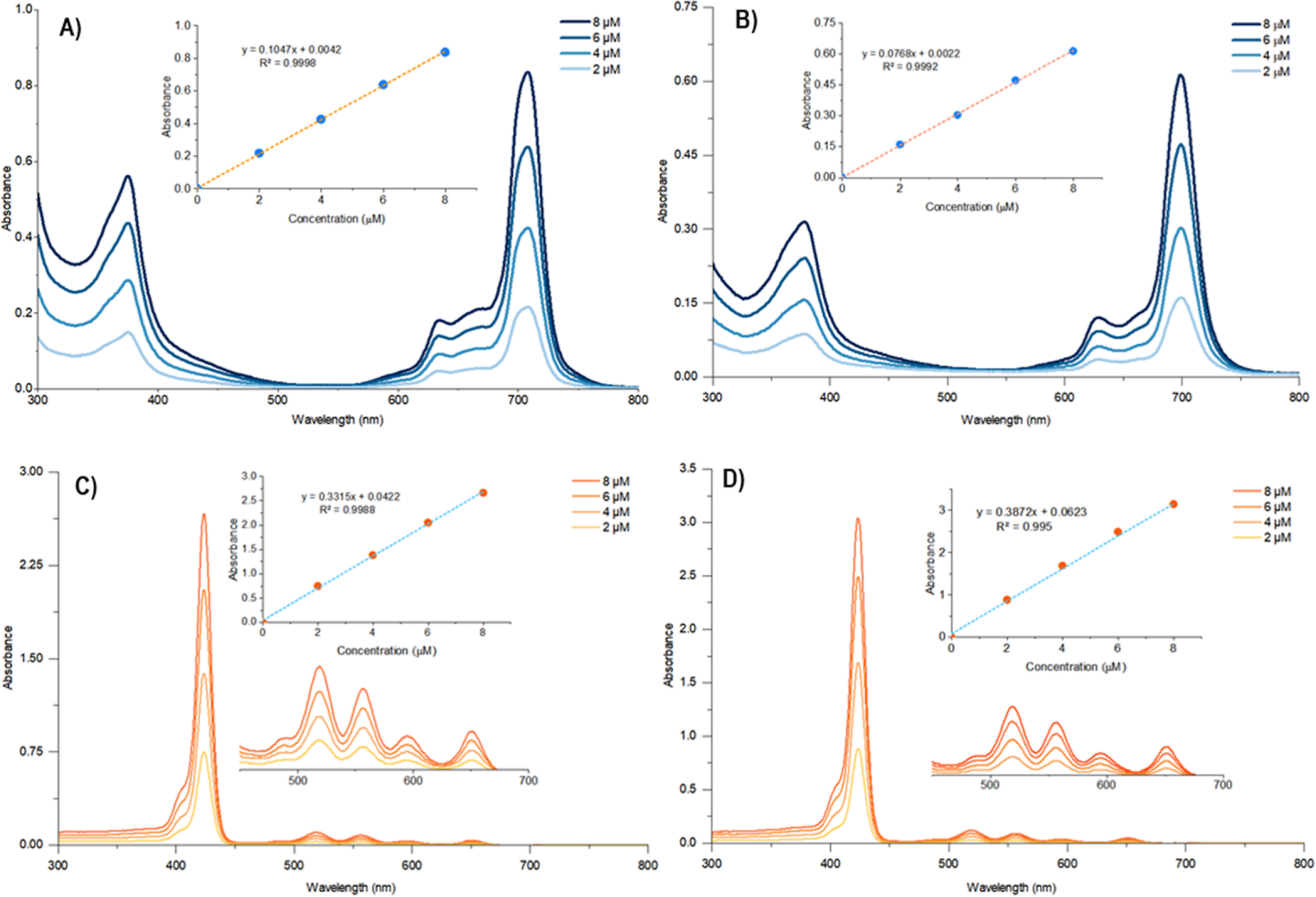
UV–vis absorption spectra of (A) **LuPcPox(OAc)**, (B) **AB**_**3**_**TPP-Pc**, (C) **AB**_**3**_**TPP-Por-C4**, and (D) **AB**_**3**_**TPP-Por-C6** in DMSO solutions of 2–8 μM concentration range. Insets: absorbance vs concentration.

In our study, the Lambert–Beer law indicating a linear relationship between absorbance and concentrations was obeyed for all porphyrin and phthalocyanine compounds in dimethylsulfoxide (DMSO) in the whole 2–8 μM concentration measurement range (Figure 2). The UV–vis spectra of macrocyclic compounds were recorded in both DMSO and tetrahydrofuran (THF), solvents in which all compounds are soluble (Figures S13–S19). Even both ring systems consist of 18 π-electrons, extensive delocalization of the π-electrons on phthalocyanine macrocycles exhibits intense Q (0, 0) band in the 600–800 nm region and a Soret (B) band between 300 and 400 nm and porphyrin derivatives exhibit strong Soret (B) bands between 415–425 nm and relatively weak four Q bands in the 500–650 nm region. This diverse absorption behavior of complexes comes from their different subunits which are isoindoles and pyrroles in the skeleton of macrocycles for phthalocyanine and porphyrin, respectively.^40,41^Table 1 summarizes the wavelengths and associated extinction coefficients in Q and Soret (B) bands in DMSO.

**Table 1:** Absorption Spectral Data of Phthalocyanines and Porphyrins in DMSO^a^

compound	Soret [nm]	*Q*_y_ (1, 0) [nm]	*Q*_y_ (0, 0) [nm]	*Q*_x_ (1, 0) [nm]	*Q*_x_ (0, 0) [nm]
**LuPcPox(OAc)**	375 (4.85)	634 (4.36)	666 (4.42)		708 (5.02)
**AB**_**3**_**TPP-Pc**	377 (4.59)	627 (4.17)	660 (4.18)		698 (4.88)
**AB**_**3**_**TPP-Por-C4**	424 (5.52)	519 (4.10)	556 (3.99)	585 (3.61)	650 (3.67)
**AB**_**3**_**TPP-Por-C6**	423 (5.58)	519 (4.17)	556 (4.06)	595 (3.68)	651 (3.79)

a The values in parenthesis refer to log ε.

When we compared absorption maxima at 708 nm for **LuPcPox(OAc)** and 698 nm for **AB**_**3**_**TPP-Pc**, TPP substitution did not contribute as an additional chromophore group on the phthalocyanine ring. The presence of the triphenylphosphonium slightly lowers the intensity of the absorbance, with log ε of 5.01 for **LuPcPox(OAc)** and 4.88 for **AB**_**3**_**TPP-Pc**; in particular, a decrease is observed when compared to the reference **ZnPc** (log ε = 5.14).

From the viewpoint of PDT, the most important aspect is the location of the λ_max_ of the band at a lower energy, shifted to longer wavelengths as far as possible. In this regard, **LuPcPox(OAc)** has the best suitability. Among all compounds, the lutetium complex **LuPcPox(OAc)** shows the most bathochromic shift of the Q-band in the absorption spectra at 709 nm^25^ in DMSO and at 705 nm in THF (Figure 2A and Tables 1 and 2). This result can be attributed to the contribution of the lanthanide metal effect.

**Table 2:** Photophysical and Photochemical Parameters of Phthalocyanine and Porphyrin PSs (2 × 10^–6^ M in THF)

compounds	λ_max_ (nm)	λ^ex^ (nm)	λ_max_^em^ (nm)	λ_max_^ex^ (nm)	log ε (660 nm)	Φ_F_	τ_F_ (ns)	Φ_Δ_
**LuPcPox(OAc)**	705	635	713		4.40	0.009	τ_1_ = 0.21 (32.47%)	0.27^24^
							τ_2_ = 3.11 (67.53%)	
**AB**_**3**_**TPP-Pc**	694	625	697	706	4.18	0.14	τ_1_ = 2.54 (35.78%)	0.75
							τ_2_ = 4.77 (64.22%)	
**AB**_**3**_**TPP-Por-C4**	420	514	660	657	3.43	0.13	8.87	0.57
**AB**_**3**_**TPP-Por-C6**	420	514	659	654	3.56	0.13	8.91	0.58
**H**_**2**_**TPP**^a^	415	514	652	650		0.13	10.3	0.58
**ZnPc**^a^	666	640	666	673		0.25^43^	2.72	0.53^47^

a Measured in this study Φ_F_: fluorescence quantum yield, τ_F_ (ns): fluorescence lifetime, Φ_Δ_: singlet-oxygen quantum yield.

For porphyrin compounds, the bathochromic shift of the Soret (B) bands have been observed at 424 nm in DMSO and at 420 nm in THF for both **AB**_**3**_**TPP-Por-C6** and **AB**_**3**_**TPP-Por-C4** compared to the reference compound **H**_**2**_**TPP** (Figures 2C,D, Tables 1 and 2). A decrease in the molar absorption coefficient intensities has been seen for porphyrins; also, log ε of 5.72 for **H**_**2**_**TPP**(42) lowers to 5.59 for **AB**_**3**_**TPP-Por-C6** and 5.51 for **AB**_**3**_**TPP-Por-C4**. In the UV–vis spectra, it was clearly observed that the most intense absorption bands (λ_max_) of all macrocycles shift bathochromically ranging from 3 to 6 nm in DMSO solutions compared to the THF solvent, which may be a result of more polarity. From the perspective of aggregation, porphyrins and phthalocyanines have a high tendency to aggregate in solution due to the π–π interaction. In our study, all structures are monomeric in DMSO and THF as well.

### Photophysics and Photochemistry

The photophysical (fluorescence quantum yield) and photochemical (singlet oxygen generation) properties of novel phthalocyanine and porphyrins were investigated in comparison with unsubstituted zinc (II)phthalocyanine (**ZnPc**) and **H**_**2**_**TPP** in THF. All the data discussed below are summarized in Table 2.

### Photophysical Properties

**Figure 3:**
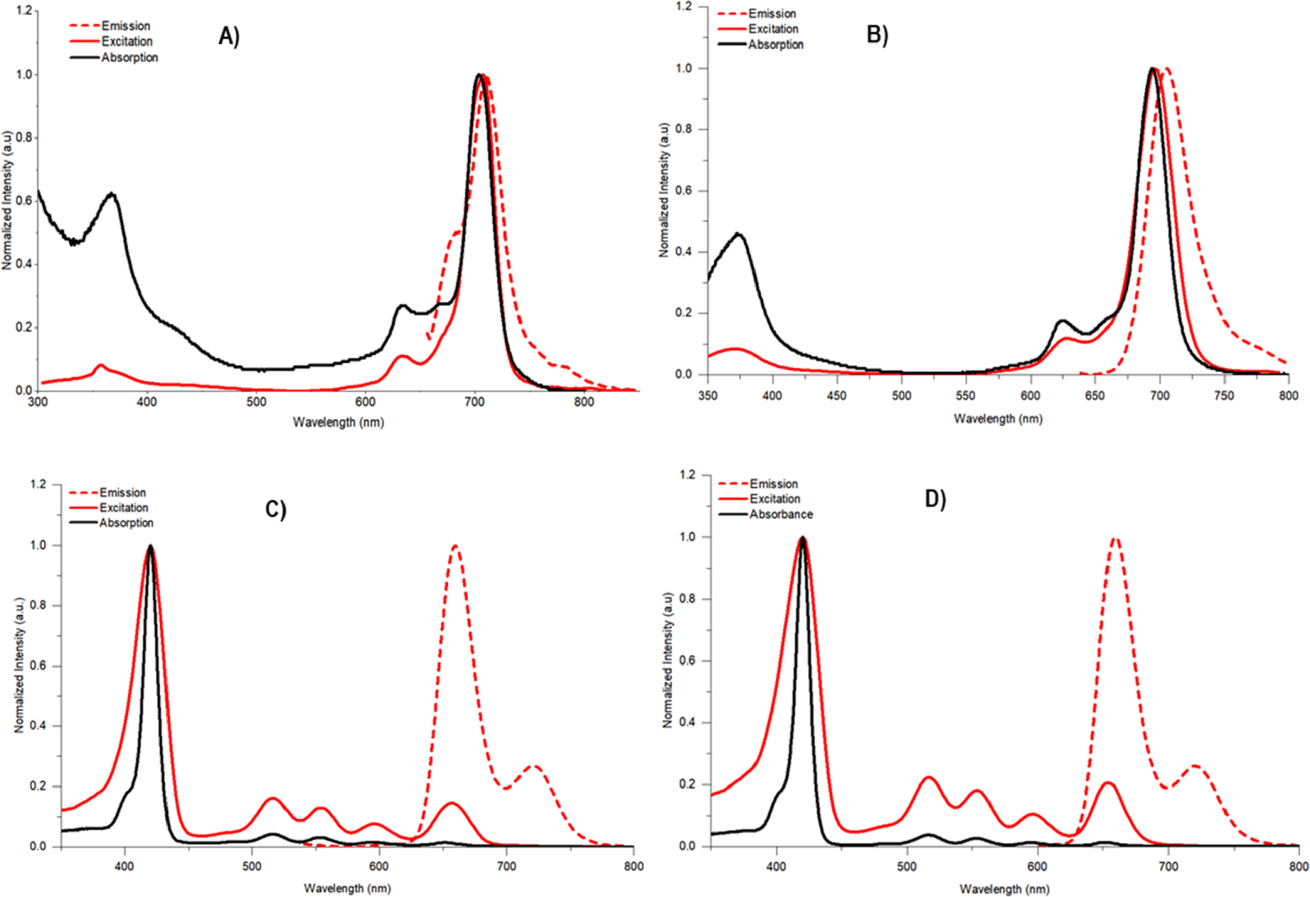
Superimposed absorption, excitation, and emission spectra of (A) **LuPcPox(OAc)**, (B) **AB**_**3**_**TPP-Pc**, (C) **AB**_**3**_**TPP-Por-C4**, and (D) **AB**_**3**_**TPP-Por-C6** in THF (λ_ex_:635, 625, 514, and 514 nm, respectively).

Excitation spectra in the Q-band region have been obtained by monitoring the fluorescence emission spectra at the indicated wavelength of porphyrins and phthalocyanine compounds. The fluorescence quantum yields (Φ_F_) of all structures were determined in THF, using a comparative method. The values were 0.009, 0.14, 0.13, and 0.13, for **LuPcPox(OAc)**, **AB**_**3**_**TPP-Pc**, **AB**_**3**_**TPP-Por-C4**, and **AB**_**3**_**TPP-Por-C6**, respectively. The lowest Φ_F_ value 0.009 belongs to **LuPcPox(OAc)** and is close to the value (0.004) obtained in DMSO in our previous study.^24^ This could be the result of the higher atomic number of lutetium in the cavity of phthalocyanine which may facilitate intersystem crossing to its excited triplet state after excitation. However, the difference and purpose of this study from our previous work are to examine whether there will be a change in the fluorescence property with the addition of the mitochondria targeting the TPP group to the structure. We achieved this goal. The Φ_F_ value of **AB**_**3**_**TPP-Pc** was 0.14, although it was lower than the reference molecule (ZnPc Φ_F=_ 0.25),^43^ and the presence of the TPP group on the structure increased the fluorescence efficiency approximately 15 times that of **LuPcPox(OAc)** Φ_F_ = 0.009. The same result was observed for porphyrin molecules with the value Φ_F_ = 0.13. Considering the porphyrin structures, it was observed that the result obtained was the same with the reference substance (**H**_**2**_**TPP** Φ_F=_ 0.13)^44,45^ which indicates that the TPP group effect did not have a positive or negative effect. The normalized absorption, excitation, and emission spectra for all compounds are shown in Figure 3, and Φ_F_ values are given in Table 2.

**Figure 4:**
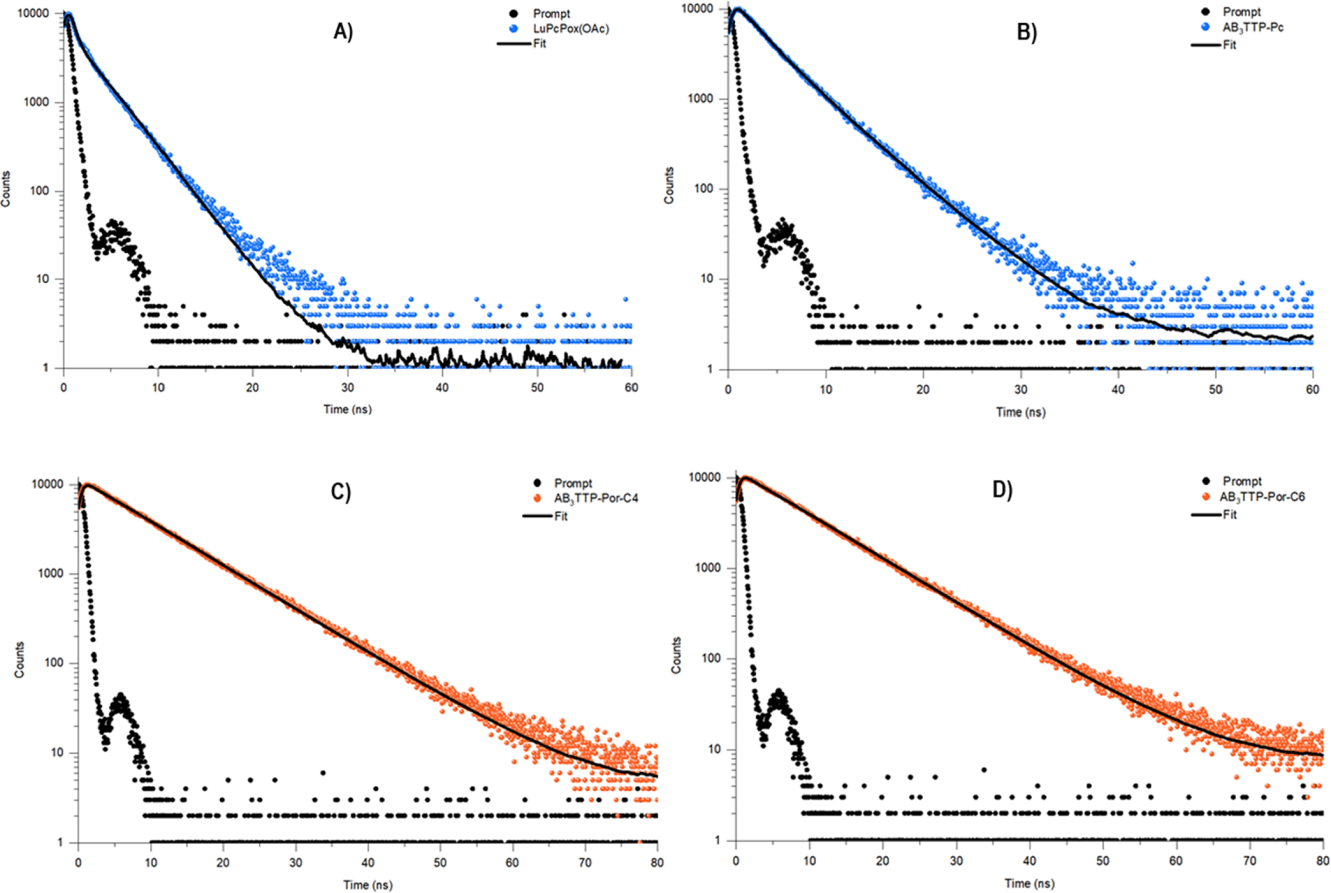
Fluorescence decay curves of (A) **LuPcPox(OAc)**, (B) **AB**_**3**_**TPP-Pc**, (C) **AB**_**3**_**TPP-Por-C4**, and (D) **AB**_**3**_**TPP-Por-C6** in THF (NanoLed λ_ex_:674 nm).

Luminescence lifetime measurements were performed on a fluorescence spectroscopy Fluorolog-3 (HORIBA Jobin Yvon) system in the single-photon counting (TCSPC) mode. A NanoLED light source (HORIBA Jobin Yvon) of λ_max_ 390 nm was used for 395 nm excitation. All measurements were carried out in 0.2 ab. solutions in THF at room temperature. The measurements of fluorescence lifetimes, as shown in Figure 4, support the photoluminescence of these molecules. **AB**_**3**_**TPP-Por-C4** and **AB**_**3**_**TPP-Por-C6** exhibit typical mono-exponential decay, **AB**_**3**_**TPP-Por-C6** has a slightly longer fluorescence lifetime (τ_f_) of 8.91 than 8.87 ns of **AB**_**3**_**TPP-Por-C4** but both less than 10.3 ns of **H**_**2**_**TPP**. **AB**_**3**_**TPP-Pc and LuPcPox(OAc)** show a biexponential decay. The short-lived species are due to component features τ_f_ of 2.54 ns (responsible for 35.78%) and τ_f_ of 2.14 ns (responsible for 32.47%) photons emitted by **AB**_**3**_**TPP-Pc** and **LuPcPox(OAc),** respectively. The longer lifetime has a τ_f_ of 4.77 ns (assume 64.22%) and 3.11 ns (assume 67.53%) of emission for **AB**_**3**_**TPP-Pc** and **LuPcPox(OAc)**, respectively.

### Photochemical Properties

For PDT, tumor cells are killed by ROS that is produced by a photosensitizer; thus, the production of ^1^O_2_ by the photosensitizer plays a vitally important role in PDT.^46^ To evaluate the photosensitizing properties of novel macrocyclic structures, singlet-oxygen quantum yields have been evaluated by a direct method in THF. Singlet-oxygen phosphorescence spectra for all compounds excited with a xenon-arc source at their respective absorption maxima were recorded by a near-IR-sensitive detector. ZnPc and H_2_TPP were used as the standard for phthalocyanine and porphyrin derivatives that have singlet oxygen yields of 0.53^47^ and 0.58^33,34^ in THF, respectively. Singlet-oxygen phosphorescence spectra of Pc and Por. derivatives in THF at equal absorbance (0.23) were obtained to directly determine Φ_Δ_ (Figures S20 and S21). As shown in Table 2, the calculated singlet-oxygen quantum yields are Φ_Δ_ = 0.27 for **LuPcPox(OAc)** and Φ_Δ_ = 0.75 for **AB**_**3**_**TPP-Pc** in THF. Apparently, **AB**_**3**_**TPP-Pc** is almost three times as efficient as **LuPcPox(OAc)** in producing singlet oxygen, matching well with our proposal that the TPP-conjugated phthalocyanine could serve as an ideal candidate for mitochondrial-targeted PDT reagent.

The ^1^O_2_ production efficiencies of porphyrin molecules were also monitored and compared by UV–vis spectra. The singlet-oxygen quantum yields are Φ_Δ_ = 0.57 for **AB**_**3**_**TPP-Por-C4** and for **AB**_**3**_**TPP-Por-C6** in THF Φ_Δ_ = 0.58, which are similar to those of the parent **H**_**2**_**TPP** (Φ_Δ_ = 0.58)^33,34^ photosensitizer. Although not very numerous,^12,32,48−50^ there is a significant number of studies on porphyrin–triphenylphosphonium conjugates in the literature. When the structure–activity relationship is examined in these studies, the influence of triphenylphosphonium-targeting groups on singlet oxygen generation is mostly similar. According to recent works, containing various numbers of targeting triphenylphosphonium groups at either the para- or meta-position,^13^ monocationic,^32,48−50^ dicationic,^35^ tricationic, or tetracationic^31^ derivatives on the porphyrin skeleton have exhibited minimum Φ_Δ_ = 0.14 and maximum Φ_Δ_ = 0.72 in different solvent conditions. As expected, **AB**_**3**_**TPP-Por-C4** Φ_Δ_ = 0.57 and **AB**_**3**_**TPP-Por-C6** Φ_Δ_ = 0.58 porphyrin derivatives exhibit nearly identical photophysical properties to the ones reported in the literature. Thus, the efficient singlet oxygen generation ability of these novel porphyrins suggests them as effective PSs for PDT application.

### Evaluating Cytotoxicity and Determining IC_50_ Values

**Figure 5:**
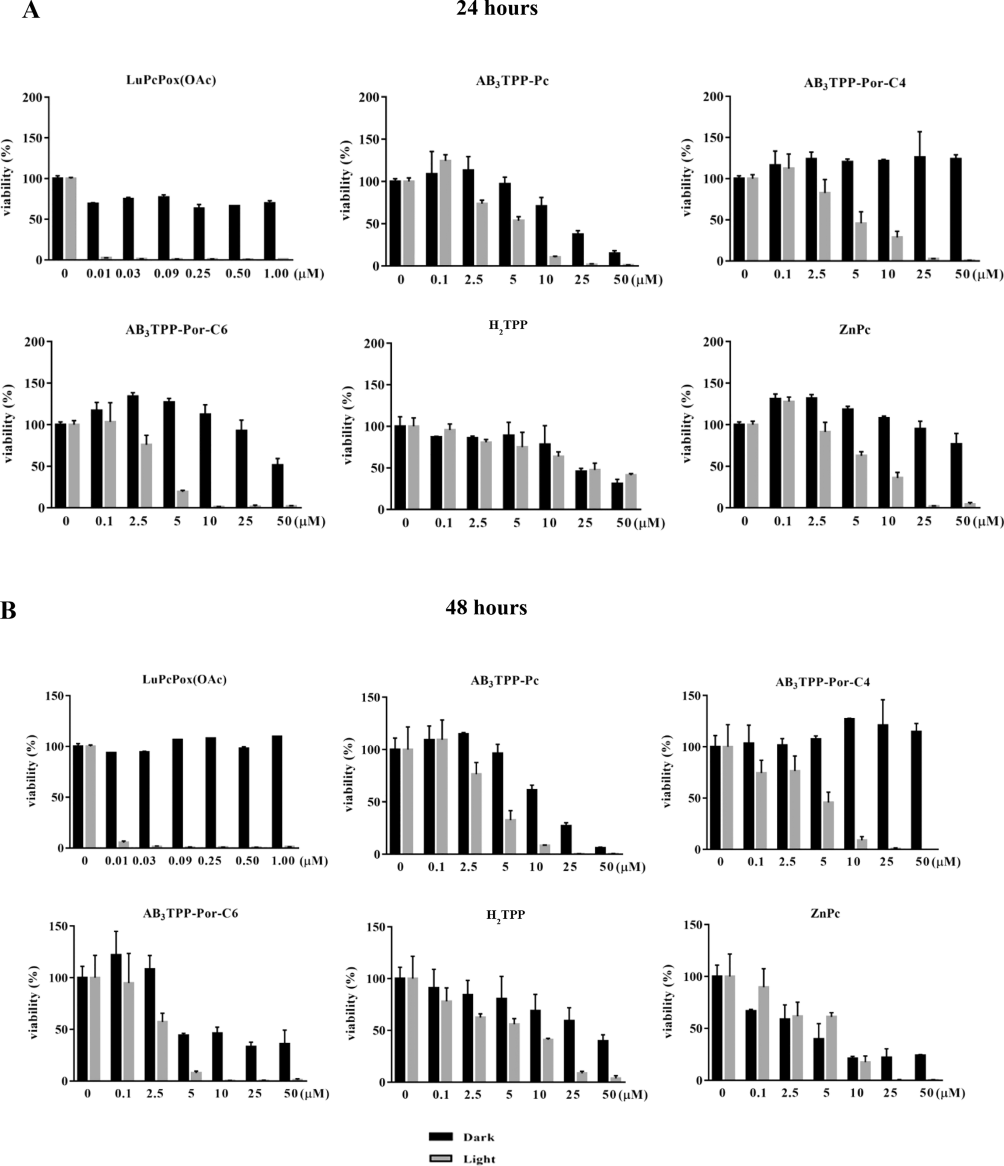
Cell viability profiles of A549 cells were evaluated at 24 (A) and 48 (B) h. The cells were incubated with a photosensitizer in the dark (black bars) and light irradiation (gray bars). The light flux was 0.036 J cm^–2^ s^–1^ for 30 min of irradiation (*n* = 4).

**Figure 6:**
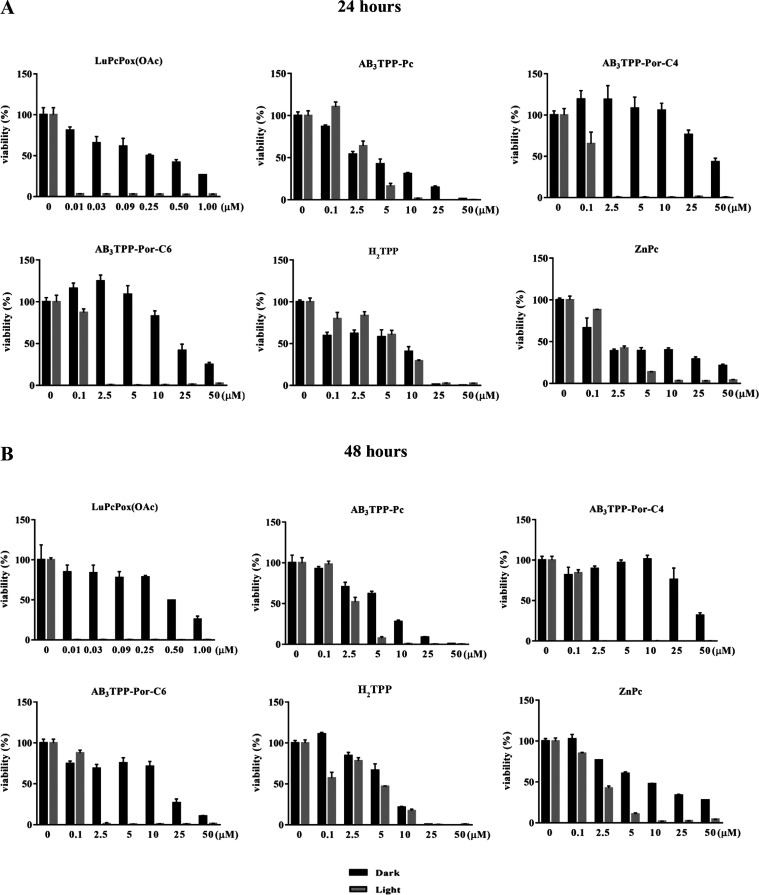
Cell viability profiles of BEAS-2B cells evaluated at 24 (A) and 48 (B) h. The cells were incubated with Pcs under dark conditions (black bars) and light irradiation (gray bars). The light flux was 0.036 J cm^–2^ s^–1^ for 30 min of irradiation (*n* = 4).

**Figure 7:**
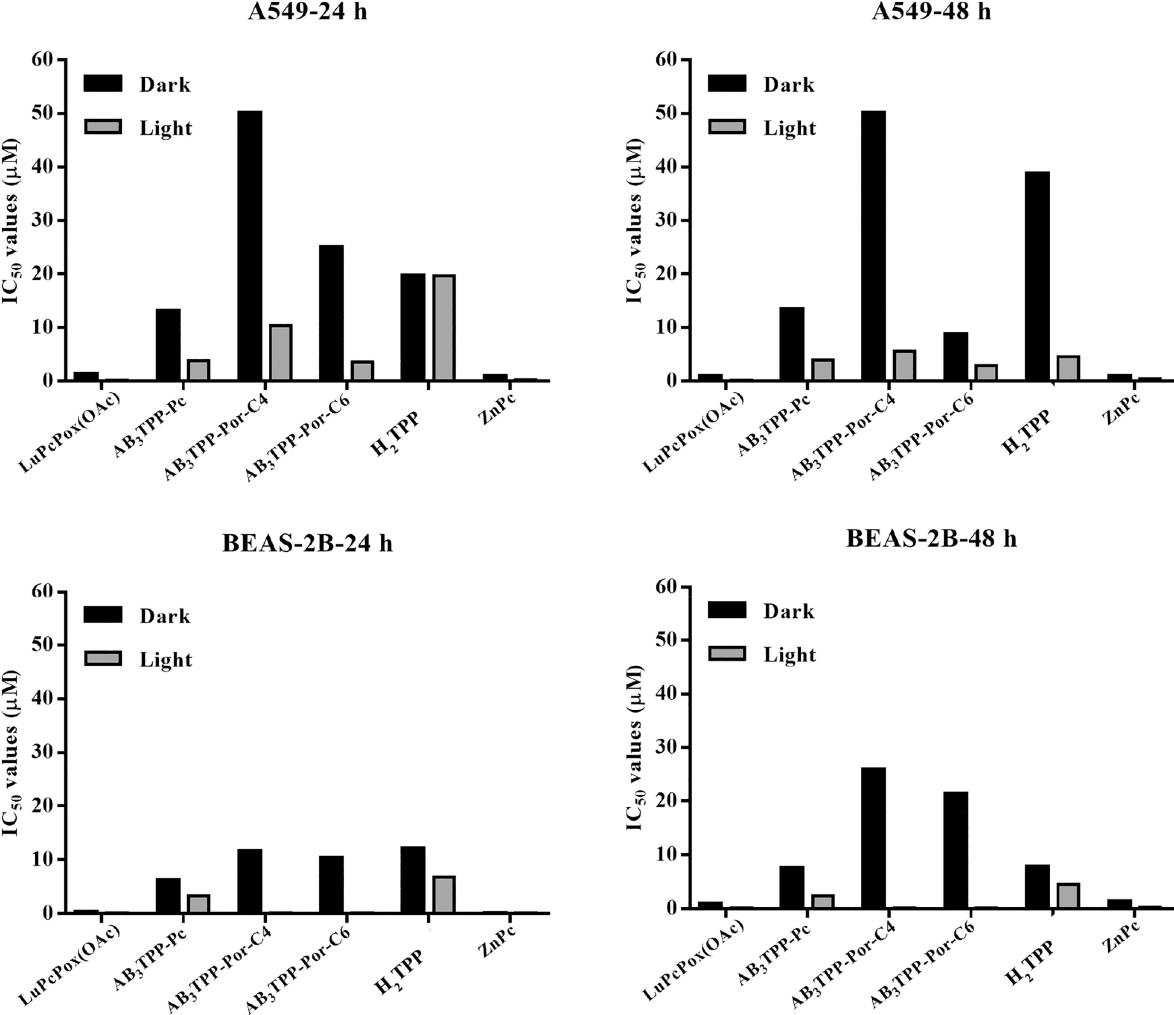
Comparative plots for IC_50_ values of all PS were collectively provided. Black columns represent the concentrations of Pcs in the dark, whereas gray columns represent the concentrations of PS under light for 24 and 48 h.

The cytotoxic effects of **AB**_**3**_**TPP-Pc**, **AB**_**3**_**TPP-Por-C4**, **PP-Por-C6**, **H**_**2**_**TPP**, and **ZnPc** molecules were evaluated at 0.1–50.0 μM concentration range for 24 and 48 h incubation time on A549 and BEAS-2B cells under dark and light conditions. **LuPcPox(OAc)** cytotoxicity was tested by the same methodology in the concentration range of 0.01–1.0 μM. Figures 5–7 represent the cell viability of A549 cells incubated with PS for 24 and 48 h. **LuPcPox(OAc)** strongly reduced the viability of the A549 cells under light exposure, and the IC_50_ value was estimated to be below 0.01 μM for 24 and 48 h incubation time (Figures 5A,B) as previously reported.^24^

The IC_50_ values of **AB**_**3**_**TPP-Pc** were approximately 13 and 4 μM for both dark and light conditions, respectively (Figures 5A,B and 7). The cells maintained their viability when treated with **AB**_**3**_**TPP-Por-C4** at high concentrations (>50 μM) under the dark condition; however, light irradiation severely reduces the viability of A549 cells for 24 and 48 h (Figures 5A,B and 7).

The viability of the A549 cells treated with **AB**_**3**_**TPP-Por-C6** and **AB**_**3**_**TPP-Por-C4** PS differs under dark conditions. The IC_50_ value of **AB**_**3**_**TPP-Por-C6** was nearly half of the IC_50_ value of **AB**_**3**_**TPP-Por-C4** without light irradiation. The A549 cells could not maintain their viability up to 48 h of **AB**_**3**_**TPP-Por-C6**. The critical concentration of **AB**_**3**_**TPP-Por**-C6 molecules for A549 cells was revealed as 2.5 μM after light irradiation, indicating a decrease in viability for both 24 and 48 h (Figures 5A,B and 7).

The viability of A549 cells after **H**_**2**_**TPP** treatment was very similar for both 24 and 48 h. The viability of A549 cells decreased in a concentration-dependent manner. The IC_50_ values were the same with and without light irradiation at 24 h. The A549 cells were viable for 48 h under dark conditions, whereas the light irradiation strongly reduced the viability of the cells (Figures 5A,B and 7). The **ZnPc** molecules reduce the cell viability of A549 cells^24^ depending on incubation time and the dosage. The viability of the cells was altered by light irradiation following **ZnPc** exposure for both 24 and 48 h of incubation time (Figures 5A,B and 7).

It can be considered from the viability graphs and IC_50_ values that BEAS-2B cells are more sensitive to PSs with or without light exposure. **LuPcPox (OAc)** treatment critically reduced the viability of BEAS-2B cells, particularly after light irradiation (Figures 6A,B and 7). Cellular response (viability) of the BEAS-2B cells was similar to A549 cells for **AB_3_TPP-Pc** exposure. **AB**_**3**_**TPP-Pc** can be considered cytotoxic at low concentrations (<6 μM) for 24 and 48 h under dark conditions. The cytotoxic concentrations are determined as 3.3 and 2.4 μM at light irradiation for **AB**_**3**_**TPP-Pc** for 24 and 48 h, respectively (Figures 6A,B and 7).

The viability values of BEAS-2B cells were lower than A549 cells for **AB**_**3**_**TPP-Por-C4** molecules. Irradiation strongly modulated the BEAS-2B viability for 24 and 48 h of incubation time (Figures 6A,B and 7). Cytotoxicity of **AB**_**3**_**TPP-Por-C6** molecules under dark conditions increased in a concentration-dependent manner. The viability of BEAS-2B cells started to decrease at 0.1 μM Pc concentration after light irradiation (Figures 6A,B and 7). The IC_50_ values for **H**_**2**_**TPP** were calculated at 12.1 μM (24 h)–7.7 μM (48 h) under dark conditions and 6.8 μM (24 h)–4.6 μM (48 h) for light exposure (Figures 6A,B and 7). About half of the BEAS-2B cells were viable at lower concentrations of **ZnPc** for 24 h with and without light. The viability of BEAS-2B cells was higher in 48 h time under dark conditions; however, light exposure induced stronger toxicity compared to 24 h incubation time.

We evaluated the cytotoxic response of A549 and BEAS-2B cells incubated with **LuPcPox(OAc)**, **AB**_**3**_**TPP-Pc**, **AB**_**3**_**TPP-Por-C4**, **PP-Por-C6**, **H**_**2**_**TPP**, and **ZnPc** molecules under the dark and light exposure conditions. The viability and IC_50_ values of the PS were tabulated in Table 3.

**Table 3:** IC_50_ Values of Phthalocyanine and Porphyrin PSs for 24 h

	IC_50_ (μM) (24 h)			
	A549		BEAS-2B	
compounds	dark	light	dark	light
**LuPcPox(OAc)**	25.6	<0.1	21.5	<0.1
**AB**_**3**_**TPP-Pc**	13.0	3.8	6.2	3.3
**AB**_**3**_**TPP-Por-C4**	>50.0	10.3	11.5	<0.1
**AB**_**3**_**TPP-Por-C6**	24.9	3.6	10.3	<0.1
**H**_**2**_**TPP**^a^	19.7	19.7	12.1	6.8
**ZnPc**^a^	>50.0	6.2	24.8	1.2

a Measured in this study.

**LuPcPox(OAc)** strongly reduced the viability of the A549 cells up to 48 h under light irradiation. The IC_50_ value under light was determined to be <0.1 μM.

The cellular responses of the A549 cells were proportionally changed by the concentration of **AB**_**3**_**TPP-Pc**. The treatment of increased **AB**_**3**_**TPP-Pc** concentration resulted in lower viability for both 24 and 48 h. **AB**_**3**_**TPP-Por-C4** may be considered as noncytotoxic under dark conditions for A549 cells up to 48 h, whereas the cells became sensitive following light irradiation. The data for the IC_50_ value of **AB**_**3**_**TPP-Por-C4** pointed out that the toxicity of the molecule increased 2-fold under light compared to the dark (Figure 7). The treatment of the A549 cells with **AB**_**3**_**TPP-Por-C6** which was another photosensitizer resulted in a severe reduction of cellular viability even for dark conditions. The MTT results for the **H**_**2**_**TPP** and **ZnPc** molecules showed similar patterns in terms of concentration. The viability of A549 cells decreased depending on the concentration of H_2_TPP, but the decrease in viability was not light-dependent. The viability for the **ZnPc** molecule demonstrated that the decrease in viability originated from light irradiation (Figure 5A,B).

The viability of BEAS-2B cells was critically decreased by **LuPcPox(OAc)** treatment. BEAS-2B cells are more sensitive than A549 cells. The effects of **AB**_**3**_**TPP-Pc** molecules on BEAS-2B cell viability were like those of A549 cells. The cytotoxic effects of **AB**_**3**_**TPP-Pc** are time-dependent for BEAS-2B cells. Cytotoxic responses of BEAS-2B cells for **AB**_**3**_**TPP-Por-C4**, **AB**_**3**_**TPP-Por-C6**, and **AB**_**3**_**TPP**-Pc suggested that these molecules were more cytotoxic. Both **H**_**2**_**TPP** and **ZnPc** molecules caused an inactivation in mitochondrion metabolism according to MTT analysis of BEAS-2B cells.

The proliferation and cell viability may be estimated by the formazan crystal formation capability of radiated/nonradiated cells. Treating the cells with PSs with or without light may result in changes in mitochondrial membrane potential. An alteration in mitochondrial potential affects the metabolic activity of the cells, suggesting that irradiation may be considered to reduce the mitochondrial activity and metabolism^51^ probably interrupting electron transfers in the electron transfer complexes in the mitochondria.

An attempt is made to link the structure to the activity (cell killing). **LuPcPox(OAc)** is the most effective PS in all the molecules studied: about 10 nM concentration of **LuPcPox(OAc)** is sufficient to kill the cells. This finding suggests that **LuPcPox(OAc)** is able to initiate ROS-induced cell death or may disrupt the cell wall efficiently. However, advanced biological studies are in need to decipher the killing mechanism. TPP-conjugated porphyrin derivatives represent similar cell-killing effects without the light, but they induce 20-fold cell killing under light exposure compared to the dark. Even though the referenced molecules **H**_**2**_**TPP** and **ZnPc** (mostly studied in the literature) have higher singlet-oxygen quantum yields (produce more ROS), their activity (the cell killing) is lower than that of the porphyrin derivatives. This finding is related to the TPP conjugation that directs the porphyrin derivatives to mitochondria, leading to mitochondrial damage. At this point, the damage mechanism should be investigated further. Another note is that at the light exposure wavelength (660 nm) phthalocyanine molecules absorb more photons (stronger extinction coefficient) compared to the porphyrin derivatives; therefore, this factor may play an important role in cell killing. The existence of a heavy atom, lutetium, might contribute to the very effective cell killing (IC_50_ < 0.01 μM) as well.

## Conclusions

Two *meso*-tetraphenylporphyrin (**AB**_**3**_**TPP-Por-C4** and **AB**_**3**_**TPP-Por-C6**) and one phthalocyanine (**AB**_**3**_**TPP-Pc**) derivatives bearing triphenylphosphonium salt-terminated alkoxy group were designed and synthesized as candidate PSs in PDT. Challenging multistep reactions produced **AB**_**3**_**TPP-Por-C4**, **AB**_**3**_**TPP-Por-C6**, and **AB**_**3**_**TPP-Pc** with reaction yields of 75, 71, and 60%, respectively. Afterward, their photophysical/photochemical properties were investigated. TPP conjugation does not change the photophysical/photochemical properties. **LuPcPox(OAc)** produces less singlet oxygen but is highly effective in cell killing. **AB**_**3**_**TPP-Por-C4**, **AB**_**3**_**TPP-Por-C6**, and **AB**_**3**_**TPP-Pc** were found to be highly cytotoxic under the light. BEAS-2B cells compared to A549 cells are more sensitive to light for TPP-conjugated molecules compared to the referenced molecules **ZnPc** and **H**_**2**_**TPP**.

Regarding the phototoxicity related to the structures, the conjugation of TPP to **AB**_**3**_**Por-C4** and **-C6** creates further toxicity compared to **H**_**2**_**TPP** for the A549 cells. **AB**_**3**_**Por-C6** reduced cell viability 5-fold compared to **H**_**2**_**TPP** 2-fold compared to **AB**_**3**_**Por-C4**. A similar phototoxicity–structure relationship is valid for BEAS-2B cells. These findings suggest structural flexibility introduced by the carbon chains helps the PS to attach mitochondrial membrane easily by the TPP substituent. There is no sufficient data to make a comparison for the phthalocyanine-based structures because their IC_50_ values are very low and have no structural similarity except the core part of the phthalocyanines. TPP-conjugated porphyrin derivatives kill the cells effectively under light. Their cell-killing activity is similar compared to **LuPcPox(OAc)** when the light absorbing factor is normalized at 660 nm: TPP-conjugated porphyrins absorb less light (lower extinction coefficients) but produce more ROS (having higher singlet-oxygen quantum yield). The singlet oxygen-producing capacity of **AB**_**3**_**TPP-Pc** is almost 3 times higher compared to **LuPcPox(OAc)** and 50% efficient with respect to ZnPc, supporting the fact that TPP-conjugated phthalocyanine could be a good photosensitizer for PDT. The high singlet oxygen generation ability of these novel porphyrin and phthalocyanine PS suggests that they might be useful for PDT applications requiring less molecule concentrations and low light flux.

## Materials and Methods

### Materials

All solvents and reagents were of reagent-grade quality and obtained commercially from Aldrich, Fluka, or Merck. Comprehensive synthesis methods and characterizations of phthalodinitrile **FN-Pox**, **FN-OH,** and **AB**_**3**_**OH-Pc, LuPcPox(OAc)** were reported in our previous work.^24^**AB**_**3**_**Br-Pc**, **AB**_**3**_**TPP-Pc**, **AB**_**3**_**Por-OH**, **AB**_**3**_**Br-Por-C4**, **AB**_**3**_**Br-Por-C6**, **AB**_**3**_**TPP-Por-C4**, and **AB**_**3**_**TPP-Por-C6** are provided in the Supporting Information (Figures S1–S21).

### Synthesis of Asymmetric AB_3_-Type TPP-Conjugated Macrocycle Ligands

#### Brominated Asymmetric (**AB**_**3**_**OH-Pc**, **AB**_**3**_**OH-Por-C4**, and **AB**_**3**_**OH-Por-C6**) Compounds

##### General Procedure for Bromine Substitution

In a reaction flask, 1 equiv of hydroxylated derivatives of **AB**_**3**_**OH-Pc** (30 mg, 0.0174 mmol), **AB**_**3**_**OH-Por-C6** (50 mg, 0.0695 mmol), and **AB**_**3**_**OH-Por-C4** (30 mg, 0.0423 mmol) and 40 equiv of 1.6-dibromohexane (0.699 mmol, 0.10 mL, 2.27 mmol, 0.42 mL) and 1,4-dibromobutane 1.69 mmol and 0.2 mL of **AB**_**3**_**OH-Por-C4** were mixed. Then, an excess of K_2_CO_3_ (250 mg, 1.74 mmol) for **AB**_**3**_**OH-Pc**, 1 g and 6.95 mmol for **AB**_**3**_**OH-Por-C6**, and 250 mg and 1.74 mmol **AB**_**3**_**OH-Por-C4** were added and stirred in dry DMF (2.5 mL) overnight at 60 °C. The reaction was carried out under an argon atmosphere. The reaction mixture was then precipitated by adding water. The remaining water phase was extracted with DCM. Water was removed in the collected DCM phase by Na_2_SO_4_. Bromine-substituted derivatives were purified by preparative thin-layer chromatography on silica gel using DCM/ethanol (15:1 for phthalocyanine, 80:1 for porphyrins) eluent systems to give 17 mg (yield: 36%), 43 mg (yield: 70%), and 30 mg (yield: 84%) of dry products. **AB**_**3**_**Br-Pc**: MALDI-TOF (matrix: DHB): calculated C_86_H_125_BrN_8_O_19_S_7_ [M]^+^: 1879.31 g/mol *m*/*z*, founded [M]^−^1878.009 g/mol. UV–vis (THF), λ_max_/nm: 695,627,378 **AB**_**3**_**Br-Por-C6**: MALDI-TOF (matrix: DHB): calculated C_53_H_47_BrN_4_O_4_ [M]^+^: 883.89 g/mol *m*/*z*, founded [M]^+^ 883.051 g/mol. UV–vis (THF), λ_max_/nm: 421, 515, 557, 598, 654 **AB**_**3**_**Br-Por-C4**: MALDI-TOF (matrix: DHB): calculated C_51_H_43_BrN_4_O_4_ [M]^+^: 855.83 g/mol *m*/*z*, founded [M]^+^ 856.397 g/mol. UV–vis (THF), λ_max_/nm: 421, 519, 553, 596, 652.

#### TPP-Conjugated Asymmetric (**AB**_**3**_**TPP-Pc**, **AB**_**3**_**TPP-Por-C4**, and **AB**_**3**_**TPP-Por-C6**) Compounds

##### General Procedure for TPP Conjugation

In a reaction flask, brominated derivatives of **AB**_**3**_**Br-Pc** (17 mg, 0.009 mmol), **AB**_**3**_**Br-Por-C6** (43 mg, 0.048 mmol), and **AB**_**3**_**Br-Por-C4** (30 mg, 0.035 mmol) were dissolved in two drops of dry DMF. It was then mixed with excess TPP (60 mg/0.23 mmol, 250 mg/0.95 mmol, 184 mg/0.7 mmol) and heated at 120° C for 16 h under an argon atmosphere, respectively. The solvent was removed under reduced pressure. The product was purified by preparative thin-layer chromatography on silica gel using a DCM/ethanol 20:1 system for phthalocyanine and 80:1 for porphyrins to give 15 mg (yield 60%), 24 mg (yield 75%), and 28 mg (yield 71%) of dry product, respectively.

**AB**_**3**_**TPP-Pc**: Fourier transform infrared (FT-IR) (ATR): ν_max_ (cm^–1^), 3292.5 (N–H), 2922.2–2866.5 (CH_2_, CH_3_), 1596 (C–N), 1259.2, 1099.8–1017.2 (C–O–C), 794.2 MALDI-TOF (matrix: DIT): calculated C_104_H_140_BrN_8_O_19_PS_7_ [M]^+^: 2138.720 g/mol *m*/*z*, founded [M – Br]^+^ 2061 g/mol. UV–vis (DMSO), λ_max_/nm: 339, 704. ^1^H NMR (500 MHz, DMSO-*d*_6_, ppm): 7.75–8.16 (m, 23H, ArH), 3.20–4.01 (m, 36H, OCH_2_ and SCH_2_), 1.22–1.49 (m, 22H, OCH_2_ and SCH_2_).

**AB**_**3**_**TPP-Por-C4**: FT-IR (ATR): ν_max_ (cm^–1^), 3318.5 (N–H), 2929.5 (CH_2_, CH_3_), 1605 (C–N), 1504.2,1438, 1244, 1173, 1107, 1030, 965, 803, 734. MALDI-TOF (matrix: DIT): calculated C_69_H_58_BrN_4_O_4_P [M]^+^: 1118.12 g/mol *m*/*z*, founded [M – Br]^+^ 1037 g/mol. UV–vis (DMSO), λ_max_/nm: 424, 519, 557, 595, 651. ^1^H NMR (500 MHz, DMSO-*d*_6_, ppm): −2.89 (s, 2H, NH), 1.23 (s, 4H, CH_2_)1.91 (b, 4H, CH_2_), 2.13 (b, 4H, CH_2_), 3.81 (m, 4H, OCH_2_), 4.06 (s, 9H, CH_2_–OCH_3_), 4.36 (t, 2H, OCH_2_), 7.32–7.40 (m, 8H, ArH-Pyrrole), 7.83–7.95 (m, 15H, ArH-TPP), 8.12 (m, 8H, ArH), 8.86 (m, 8H, ArH).

**AB**_**3**_**TPP-Por-C6**: FT-IR (ATR): ν_max_ (cm^–1^), 3314.8 (N–H), 2929.6 (CH_2_, CH_3_), 1604 (C–N), 1500,1437, 1244, 1171, 1106, 1032, 964, 801, 721. MALDI-TOF (matrix: DHB): calculated C_71_H_62_BrN_4_O_4_P [M]^+^: 1146.18 g/mol *m*/*z*, founded [M – Br] ^+^ 1065 g/mol. UV–vis (DMSO), λ_max_/nm: 423, 519, 557, 595, 651. ^1^H NMR (500 MHz, DMSO-*d*_6_, ppm): −2.88 (s, 2H, NH), 1.65 (s, 8H, CH_2_)1.85 (b, 2H, CH_2_), 3.67 (m, 4H, OCH_2_), 4.03 (s, 9H, CH_2_–OCH_3_), 4.20 (m, 2H, OCH_2_), 7.40 (m, 8H, ArH-Pyrrole), 7.81 (m, 15H, ArH-TPP), 8.09 (m, 8H, ArH), 8.54 (m, 8H, ArH).

### Cell Culture and Treatments

A549 and BEAS-2B cells were obtained from ATCC with reference numbers CCL-185 and CRL-9609, respectively. A549 cells were cultured in DMEM (with 2 mM l-glutamine, Gibco, United States), while RPMI 1640 (with 2 mM l-glutamine, Gibco, United States) was used for BEAS-2B cells. Both media were supplemented with 10% fetal bovine serum (FBS) (Gibco, United States), and cells were cultured in an incubator with a humidified atmosphere of 5% CO_2_ at 37 °C.^24^

### Cell Viability

For the evaluation of cytotoxic activity, an MTT assay was employed. Cells were seeded 1 day prior to treatment in 96-well plates at a concentration of 20,000 cells/mL. Followed by overnight incubation, the cells were exposed to **LuPcPox(OAc)**, **AB3TPP-Pc**, **AB3TPP-Por-C4**, **PP-Por-C6**, **H**_**2**_**TPP**, and **ZnPc** molecules. All molecules were dispersed in DMSO. The working concentrations of the molecules contain <0.1% (v/v) of DMSO. Time-point assays were performed after 24 and 48 h. The final concentration of MTT was 0.5 mg/mL, and after 3 h incubation, absorbances were measured at 545 nm. The absorbance of the blank group which was the samples containing media with 10% FBS was subtracted from the measurements and cell viability was determined by the following formula.^52^ The control group was composed of nontreated cells. Cell viability (%) = (absorbance value of sample/absorbance value of control) × 100.

### Irradiation of the Cells

The cells seeded at a concentration of 20,000 cells/mL in the 96-well plates were irradiated at 660 nm using an LED light array producing a flux of 0.036 J cm^–2^ s^–1^ (0.036 W/cm^2^). In order to determine the cytotoxic effects of Pcs under light, one group of each cell line was exposed to an LED array for 30 min at room temperature and then returned to the incubator, while another group was incubated in the dark.^24^ The delivered red photons on the cells were measured by a calibrated PAR meter (Apogee SQ-520). The temperature of the chamber under continuous light flux was monitored synchronously for ensuring the stability of the conditions.^53^

The cells were seeded at a concentration of 20,000 cells/mL on 96-well plates and incubated for 24 h. The Pcs were added in 0.1, 2.5, 5, 10, 25, and 50 μM concentrations for evaluating the toxicity with/without light except for **LuPcPox(OAc)**. The **LuPcPox(OAc)** was added into the wells as 0.01, 0.03, 0.09, 0.25, 0.50, and 1 μM concentration because a concentration over 1.0 μM kills the cells immediately. The Pcs in the cell media were discarded, and a fresh medium was added to the cells to remove noninternalized molecules. The viability of the cells was recorded for 24 and 48 h after the addition of Pcs. The cells were irradiated for 30 min.

IC_50_ values of the Pcs were determined by fitting the cell’s viability curve acquired from the spectroscopic data. All the values were obtained by using Origin Software (Figure 7).

## Supporting Information Available

General information related to reagents, solvents, methods, spectroscopic techniques, and photophysical and photochemical measurements; FT-IR, NMR, and mass spectra of compounds; UV–vis spectra of porphyrin and phthalocyanine derivatives; and Singlet-oxygen Phosphorescence data (PDF)
