# Supplementary material for: Development of AB3-Type Novel Phthalocyanine and Porphyrin Photosensitizers Conjugated with Triphenylphosphonium for Higher Photodynamic Efficacy
Source: ACS Omega. 2022 Oct 19;7(43):39404–16. doi: 10.1021/acsomega.2c05814 (PMC9631755; doi:10.1021/acsomega.2c05814)
Supplement: Supplementary file 1 — ao2c05814_si_001.pdf [file ao2c05814_si_001.pdf]

Development of AB_3 Type Novel Phthalocyanine and Porphyrin Photosensitizers Conjugated with Triphenylphosphonium for Higher Photodynamic Efficacy

Emel Önal[a,b], Özge Tüncel[c, d] , İpek Erdoğan Vatansever[d], Mohamad Albakour[a], Gizem Gümüşgöz Çelik[a], Tuğba Küçük[a], Bünyamin Akgül[d], Ayşe Gül Gürek[a*], Serdar Özçelik[c*],

[a] *Gebze Technical University, Department of Chemistry, Gebze, 41400 Kocaeli, Turkey*

[b] *Doğuş University, Faculty of Engineering, Ümraniye, 34775 Istanbul, Turkey*

[c] *Izmir Institute of Technology, Faculty of Science, Department of Chemistry, Urla, 35430 Izmir, Turkey*

[d] *Izmir Institute of Technology, Faculty of Science, Department of Molecular Biology and Genetics, Urla, 35430 Izmir, Turkey*

Supporting Information

Table of Contents…………………………………………………………………………………. Page

1. General Information……………………………………………………………………………... S2

2. FT-IR, NMR and Mass Spectra of Compounds………………………………………………… S4

4. Absorption Spectroscopy……………………………………………………………………… S10

5. Singlet Oxygen Phosphorescence Data……………………………………………………….. S13

5. References……………………………………………………………………………………… S14

1. General Information

Reagents and solvents: All reagents, purchased from fine chemical suppliers Aldrich, Merck, Alfa Aesar, and Fluka, were used without further purification unless otherwise stated. Solvents were either used as commercially supplied or used as purified by standard techniques.

Methods : Nonaqueous reactions were performed in oven-dried glassware under argon atmosphere at reported temperature. Reactions were monitored by thin layer chromatography on Merck silica gel 60 F254 precoated aluminum sheets. Spots were visualized either by UV 254 nm light. Column chromatography was performed on Merck silica gel 60 (230-400 mesh size) or preparative TLC (silica gel) using the appropriate solvent system as eluent.

Infrared Spectroscopy : Infrared spectra were recorded on a PerkinElmer Spectrum 100 FT-IR spectrometer with an attenuated total reflection (ATR) accessory featuring diamond/ZnSe plate. Samples were deposited as neat on ATR module and absorption maxima (ν_{max}) were quoted in wavenumbers (cm^{-1}) in the range of 4000-650 cm^{-1}. Intensities of absorptions are abbreviated as s, strong; m, medium; and w, weak.

NMR Spectroscopy : ^{1}H and ^{13}C nuclear magnetic resonance spectra were taken on Bruker Ultrashield Plus 500 MHz FT-NMR spectrometer. Deuterated solvents were used in all NMR measurements. Chemical shifts (δ) are reported in parts per million (ppm) relative to residual solvent peaks as an internal reference for ^{1}H-^{13}C nuclei. Assignments were determined on the basis of either unambiguous chemical shift or coupling patterns. Peak multiplicities are defined as s = singlet, d = doublet, t = triplet, q = quartet, m = multiplet, br = broad.

Mass Spectra : Matrix-assisted laser desorption ionization time-of-flight (MALDI-TOF) mass spectra were recorded on a BRUKER Microflex LT spectrometer. The matrix is indicated in brackets for each compound.

Absorption Spectroscopy : Absorption spectra were recorded at room temperature on a Shimadzu UV-2600 UV-vis and UV-VIR spectrometer using a 1 cm path length quartz cuvette between maximum range 300 and 1400 nm. Solutions were prepared in spectro-grade solvents. Molar extinction coefficients (ε) were determined by measurement of the absorption of t five solutions of differing concentration for each radical, followed by determination of the slope.

Photophysical and Photochemical Measurements: Steady-state fluorescence excitation and emission spectra were recorded by using a Varian Cary Eclipse spectrofluorometer using 10 mm path length cuvettes at room temperature. ***Fluorescence quantum yield (ϕ_F) determination.*** Fluorescence quantum yield values (ϕ_F) were determined by using William's method[1]. Accordingly, the UV-Vis absorbance and fluorescence emission spectra at different concentrations of the reference standard (**ZnPc**) and samples were measured under identical conditions. Integrated fluorescence intensities versus absorbance for **ZnPc** (ϕ_F= 0.25 in THF[2]) and **LnPcs** were plotted. Slopes of the plots were proportional to the quantity of the quantum yield. Equation 1 was used to calculate quantum yield (ϕ_F) values. In the equation, Grad is the gradient of the plot and *n* is the refractive index of the solvent used for samples and standards.

$$\Phi_F = \Phi_F^{Std}\left(\frac{Grad}{Grad_{Std}}\right)\left(\frac{n^2}{n_{Std}^2}\right) \quad (1)$$

Singlet oxygen quantum yield (Φ_Δ) determination. For indirect method, singlet oxygen quantum yield (Φ_Δ) determinations were carried out a 1 mL portion of the solutions containing the singlet oxygen quencher irradiated in the Q band region with the photo-irradiation set-up described in the reference[3]. For singlet oxygen measurements, DPBF was used as chemical quencher (Φ_Δ **ZnPc** = 0.53 in THF[4]). DPBF degradation at 417 nm was monitored by UV-Vis spectroscopy. The solutions of 1×10^{-5} M of **LnPc** complexes (2.5 mL) containing DPBF 1 mL (~1×10^{-5} M) were prepared in the dark and irradiated in the Q band region. The light intensity of 7.05×10^{15} photons s^{-1} cm^{-2} was used for Φ_Δ determinations. Singlet oxygen quantum yields (Φ_Δ) were calculated using the equation 2. In the equation, R and R_{Std} were DPBF's photobleaching rates in the presence of the respective samples and standard, respectively, while I_{abs} and I_{abs}^{std} were the rates of light absorption by the samples and standard, respectively.

$$\Phi_\Delta = \Phi_\Delta^{Std}\frac{R\cdot I_{Abs}^{Std}}{R^{Std}\cdot I_{Abs}} \quad (2)$$

Photo-irradiations were studied using a General Electric Quartz line lamp (300W). A 600 nm glass cut off filter (Schott) and a water filter were used to filter off ultraviolet and infrared radiations, respectively. An interference filter (Intor, 670 nm with a band width of 40 nm) was additionally placed in the light path before the sample. Light intensities were measured with a POWER MAX5100 (Molelectron detector incorporated) power meter.

For direct method, the calculation of the $^1O_2$ quantum yields was based on the detection of NIR $^1O_2$ luminescence by an optical method based on the comparison of single molecular oxygen phosphorescence produced by the Pc sample with that generated by the reference ZnPc in the near infrared region at 1276 nm. Φ_Δ values were calculated according to Equation 3.

$$\phi_{\Delta s} = \phi_{\Delta r}\frac{\eta_s^2 A_r I_s}{\eta_r^2 A_s I_r} \quad (3)$$

In Eq. 3, $\Phi_{\Delta s}$ and $\Phi_{\Delta r}$ are the quantum yields of the sample and reference and η_s and η_r are refractive indexes of the solvents used for the measurements of the sample and reference. A_s and A_r are the absorbance of the sample and the reference, and I_s and I_r are the integrated areas under the emission spectra of the sample and the reference, respectively. The measurements were done by Horiba Jobin-Yvon Fluorometer with Hamamatsu NIR PMT 5509.

2. FT-IR, NMR and Mass Spectra of Compounds

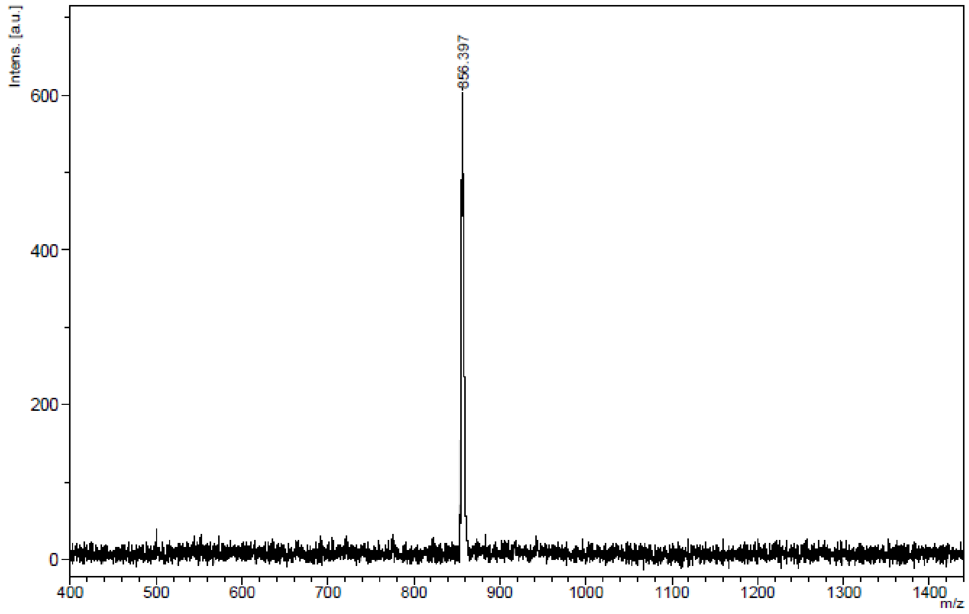

m/z	SN	Quality Fac.	Res.	Intens.	Area
856.397	32.3		1528	601.65	587

Figure S1 MALDI-Mass spectrum of **AB$_3$Br-Por-C4**

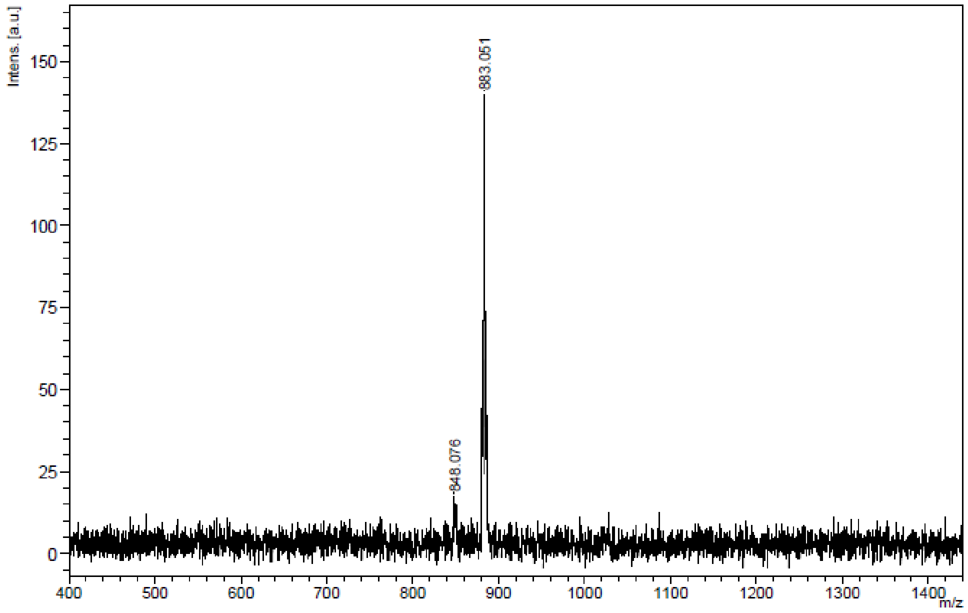

m/z	SN	Quality Fac.	Res.	Intens.	Area
848.076	2.3		1873	17.30	10
883.051	18.3		1500	139.91	158

Figure S2 MALDI-Mass spectrum of **AB$_3$Br-Por-C6**

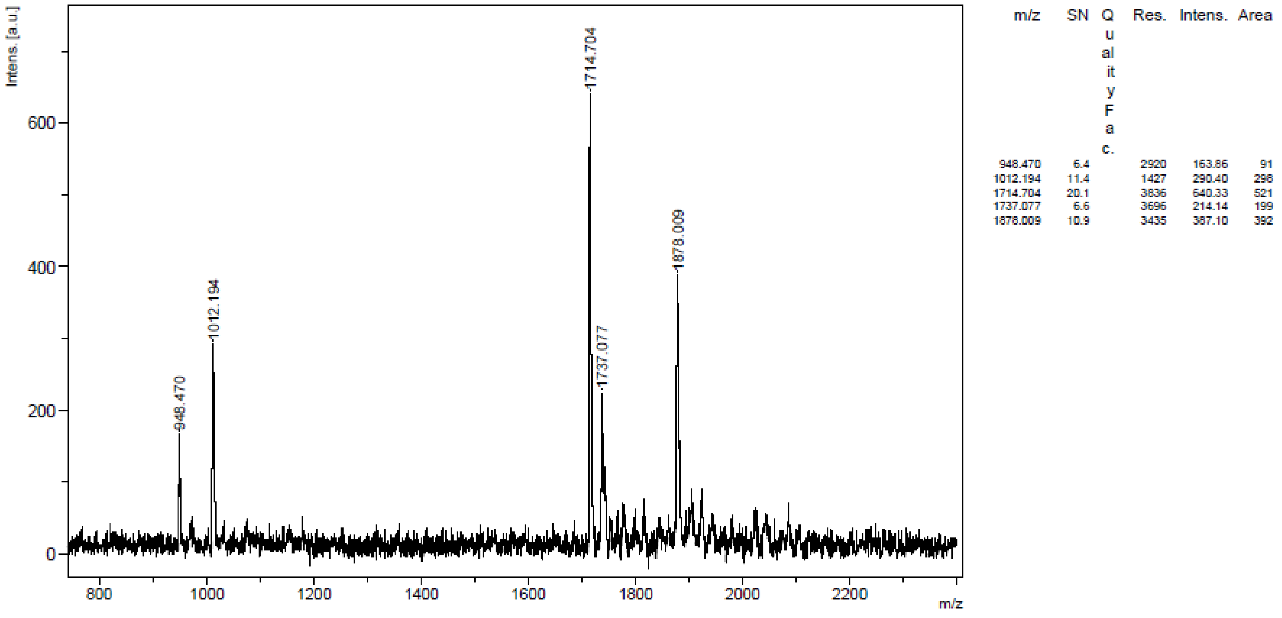

Figure S3 MALDI-Mass spectrum of **AB3Pc-Br**

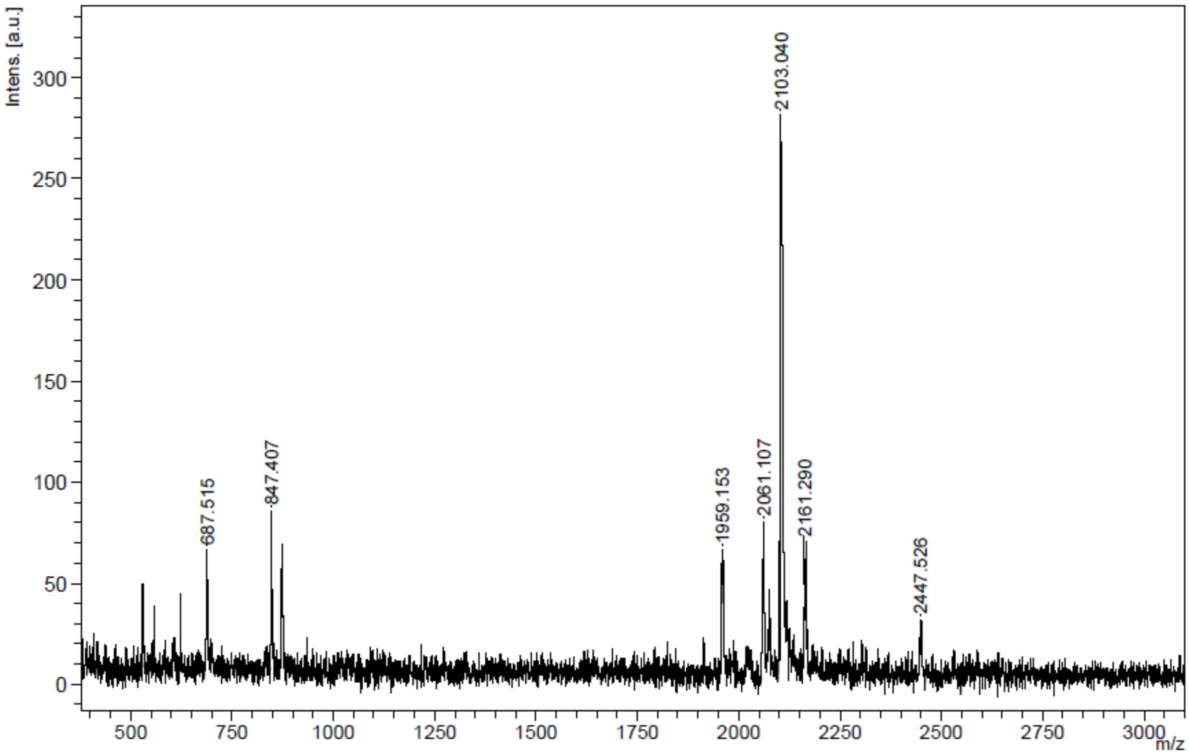

Figure S4 MALDI-Mass spectrum of **AB3TPP-Pc**

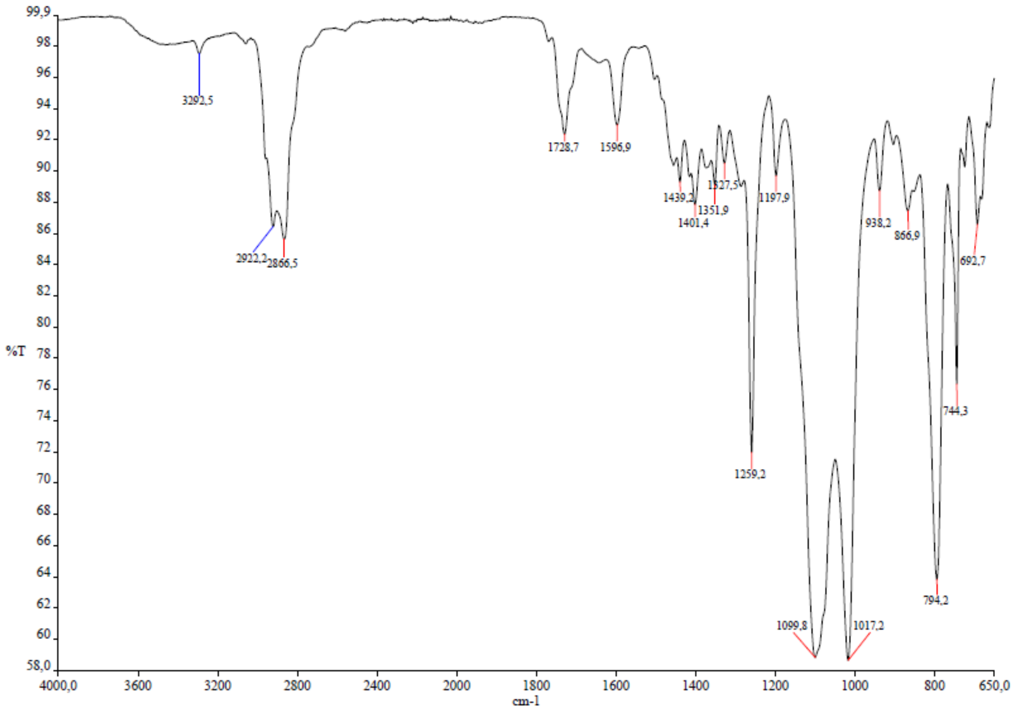

Figure S5 FT-IR spectrum of **AB$_3$TPP-Pc**

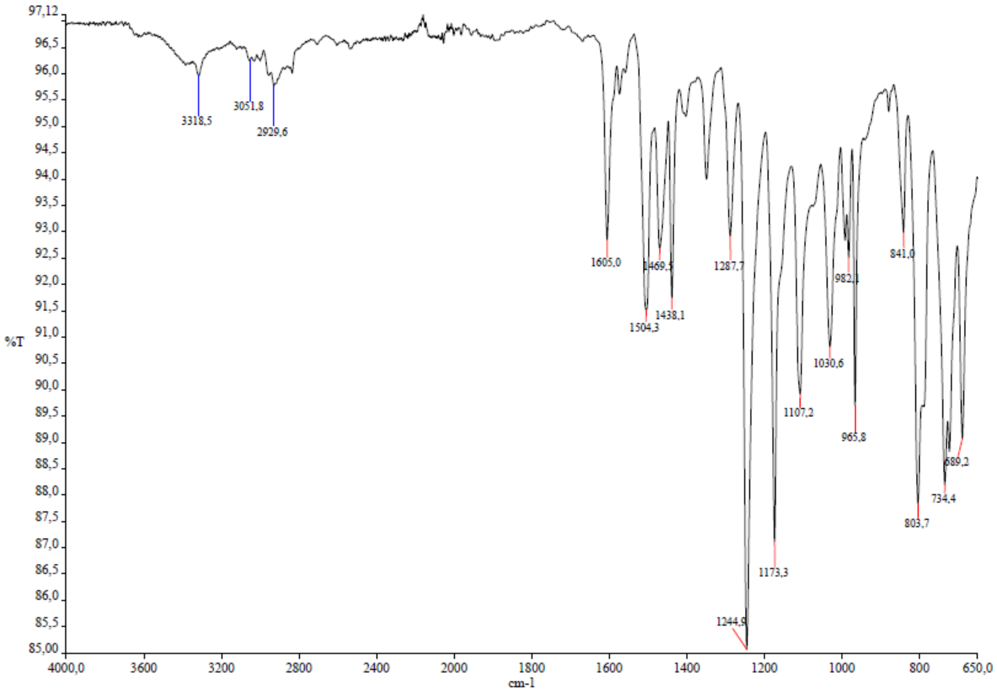

Figure S6 FT-IR spectrum of **AB$_3$TPP-Por-C4**

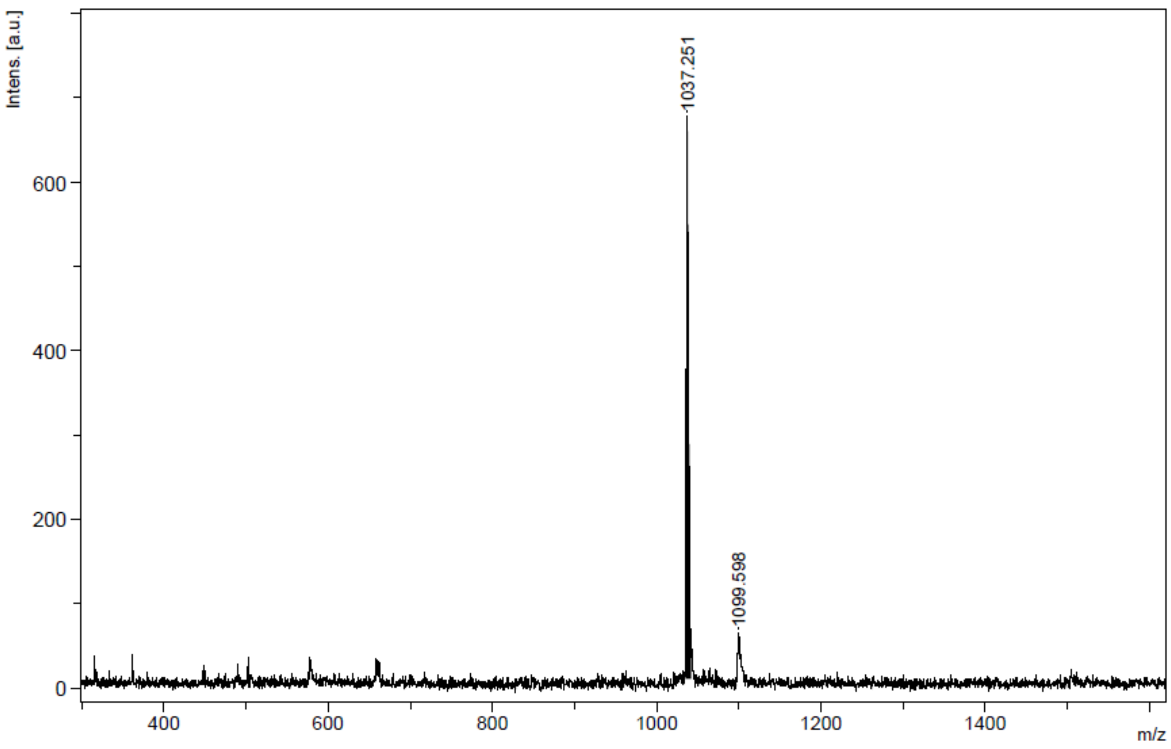

Figure S7 MALDI-Mass spectrum of **AB3TPP-Por-C4**

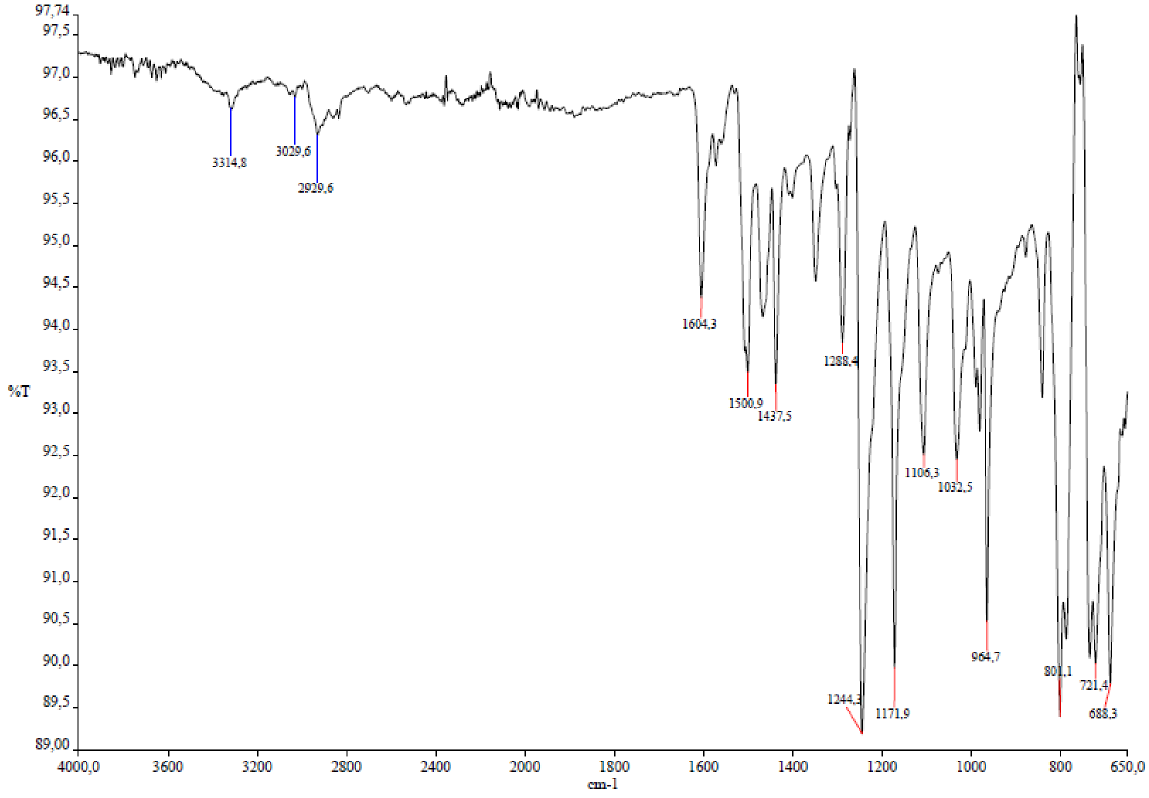

Figure S8 FT-IR spectrum of **AB3TPP-Por-C6**

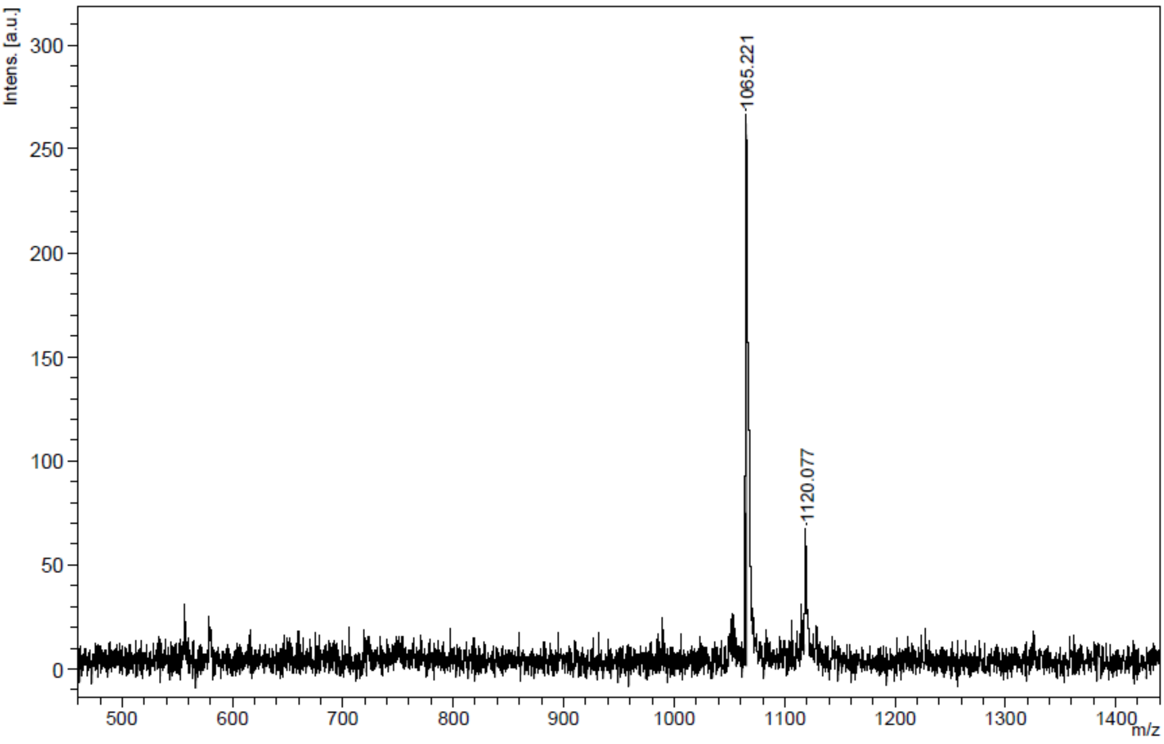

Figure S9 MALDI-Mass spectrum of **AB3TPP-Por-C6**

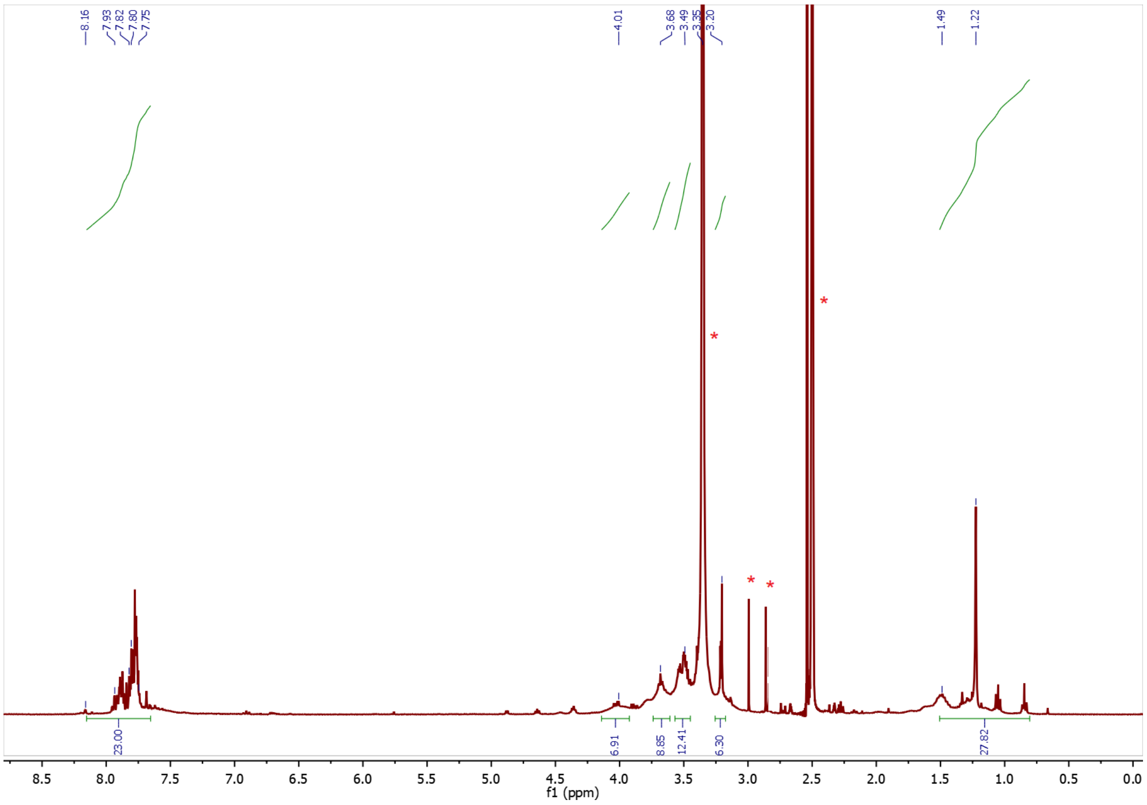

Figure S10 ^{1}H NMR spectrum of **AB3TPP-Pc** in d_6-DMSO (* solvent impurities)

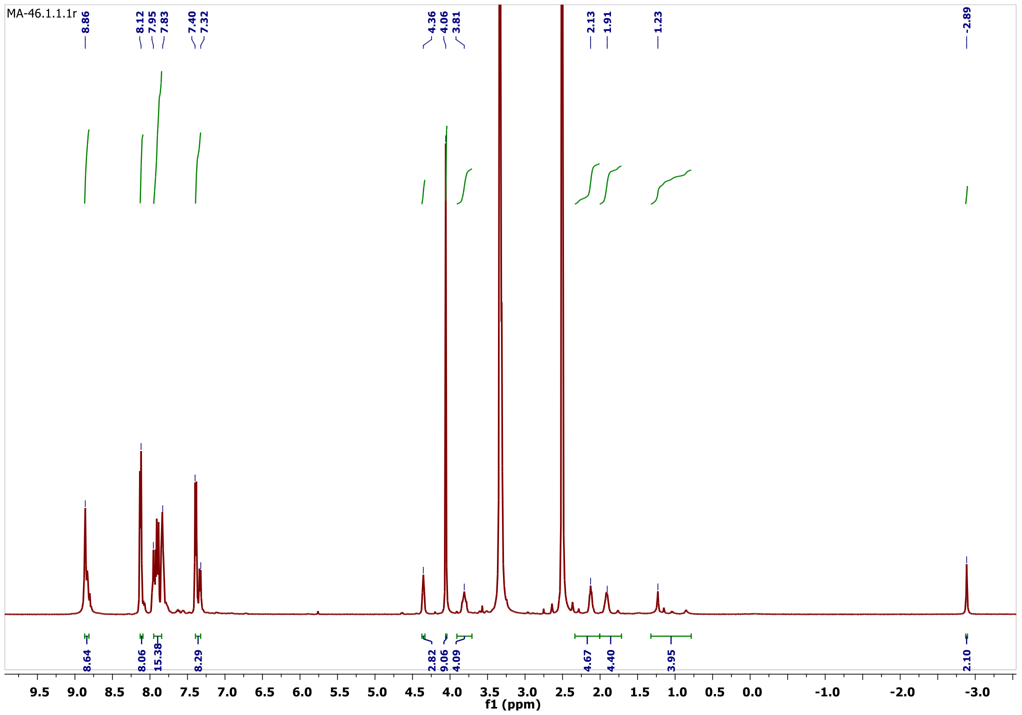

Figure S11 ^{1}H NMR spectrum of **AB$_3$TPP-Por-C4** in *d*$_6$-DMSO

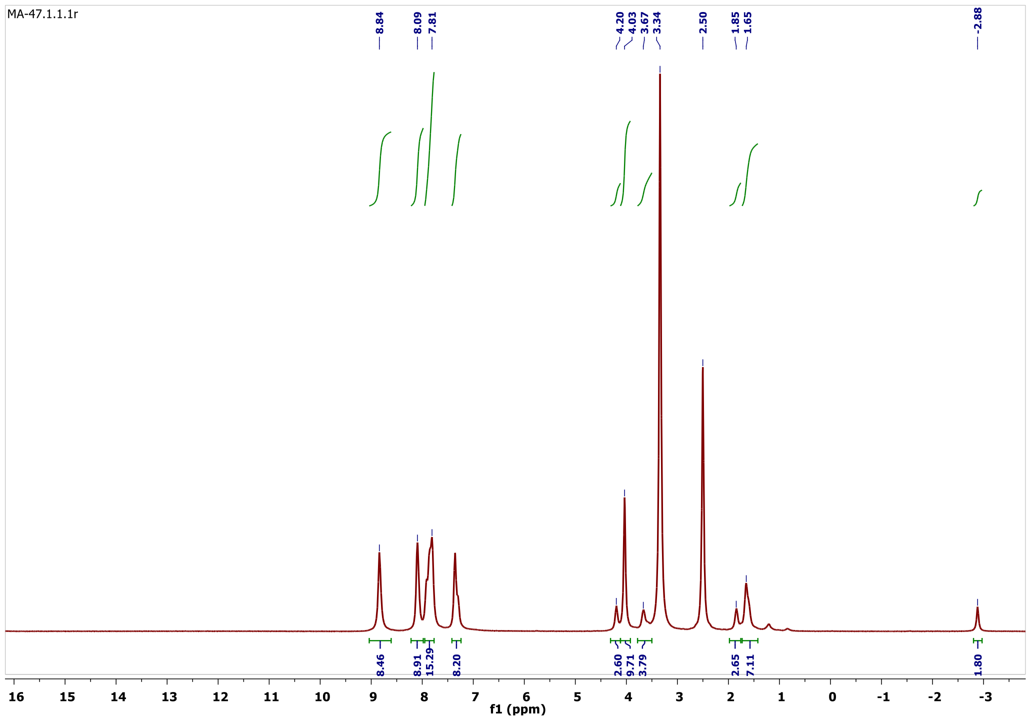

Figure S12 ^{1}H NMR spectrum of **AB$_3$TPP-Por-C6** in *d*$_6$-DMSO

3. Absorption Spectroscopy

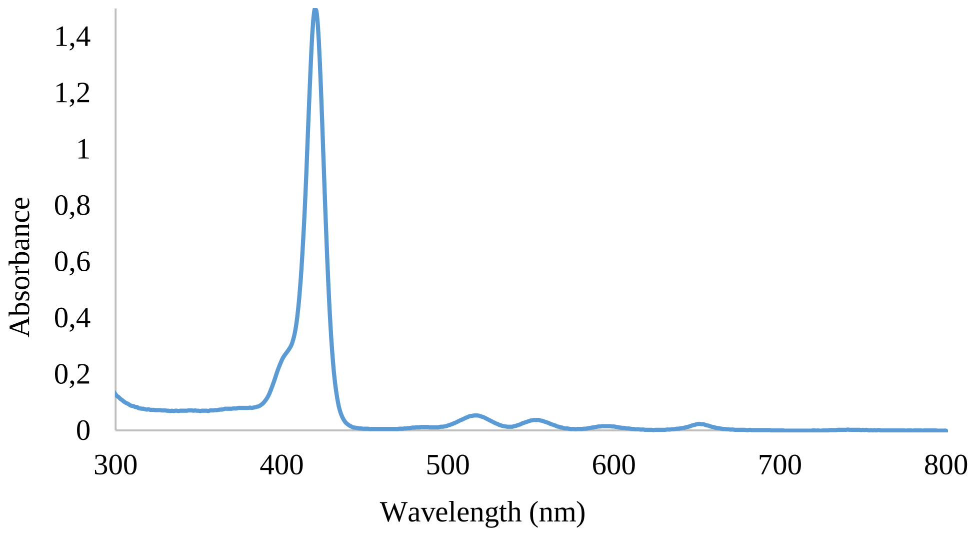

Figure S13 UV–Vis spectrum of **AB$_3$OH-Pc** in DMSO.

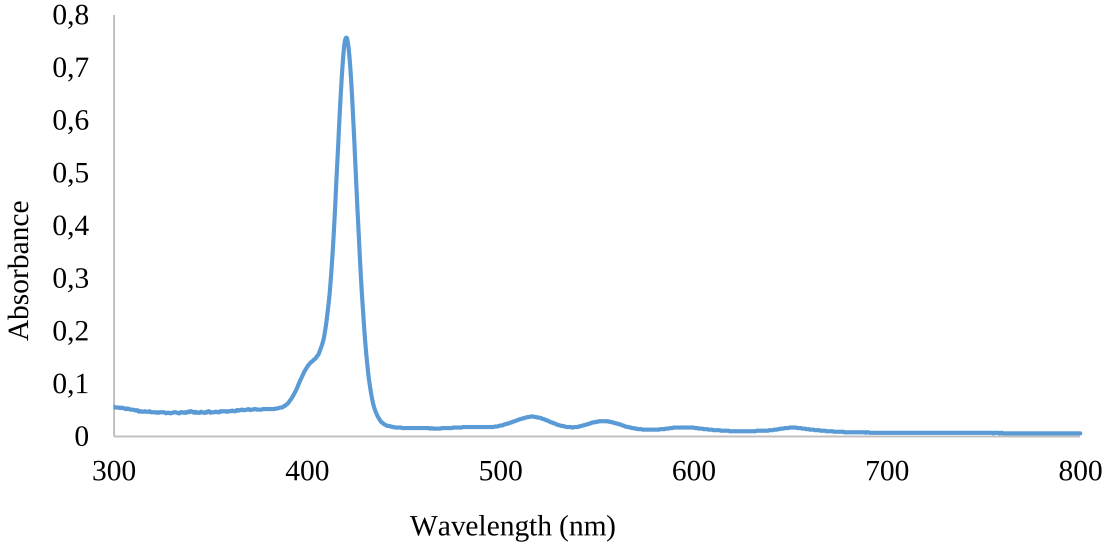

Figure S14 UV–Vis spectrum of **AB$_3$Br-Por-C4** in THF.

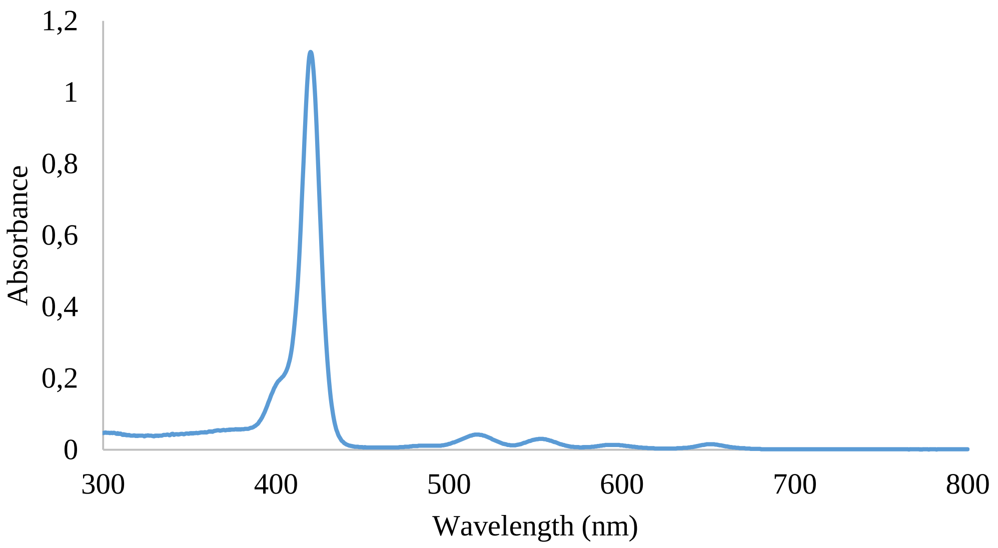

Figure S15 UV–Vis spectrum of **AB_3Br-Por-C6** in DMSO.

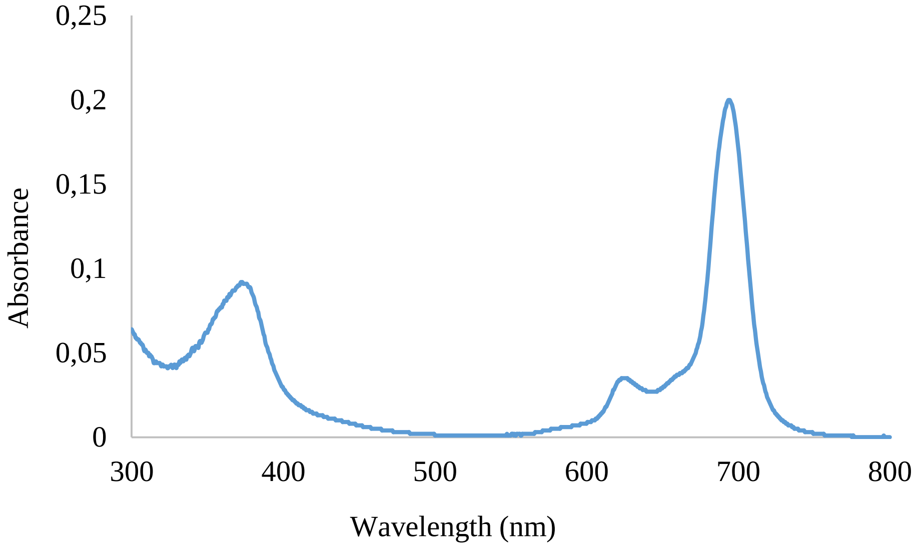

Figure S16 UV–Vis spectrum of **AB_3Br-Pc** in DMSO.

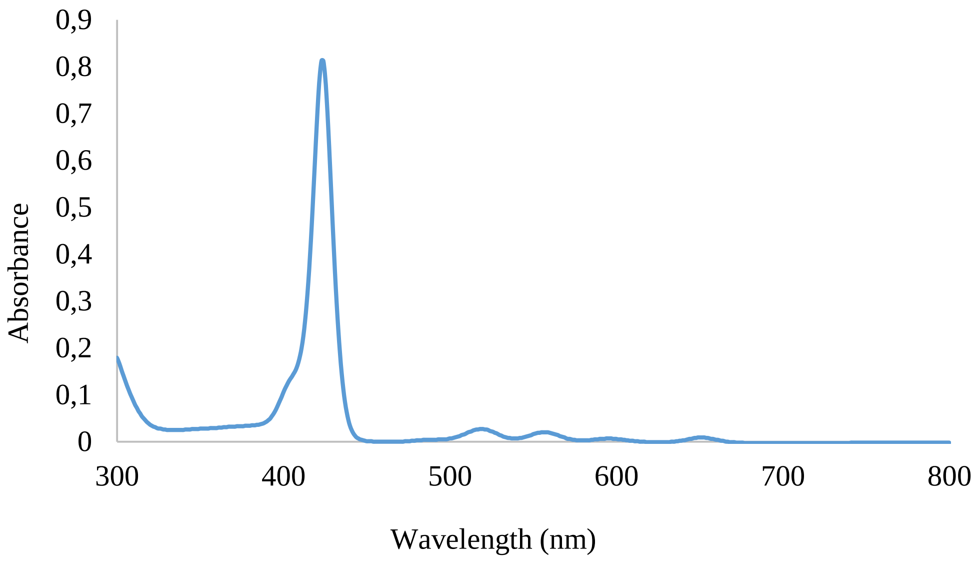

Figure S17 UV-Vis spectrum of **AB$_3$TPP-Por-C4** in DMSO

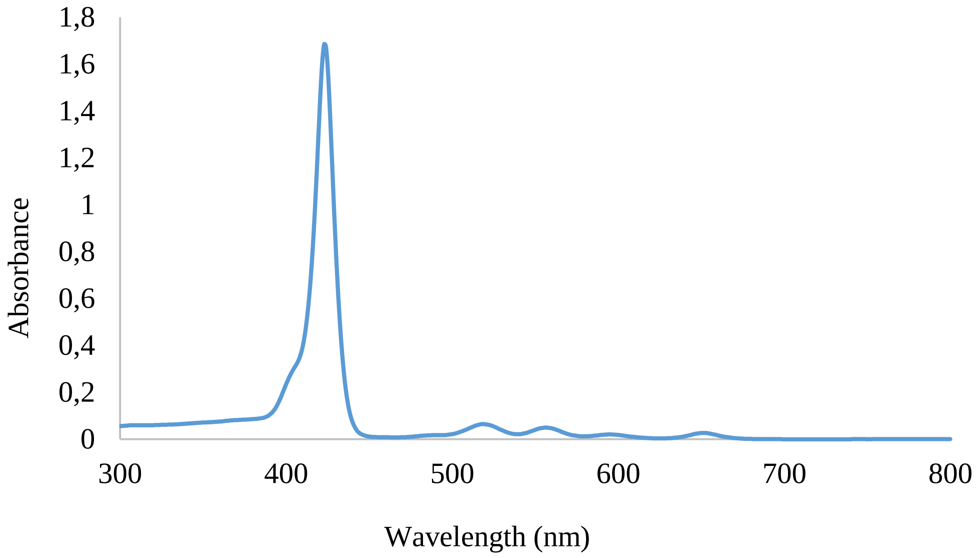

Figure S18 UV-Vis spectrum of **AB$_3$TPP-Por-C6** in DMSO

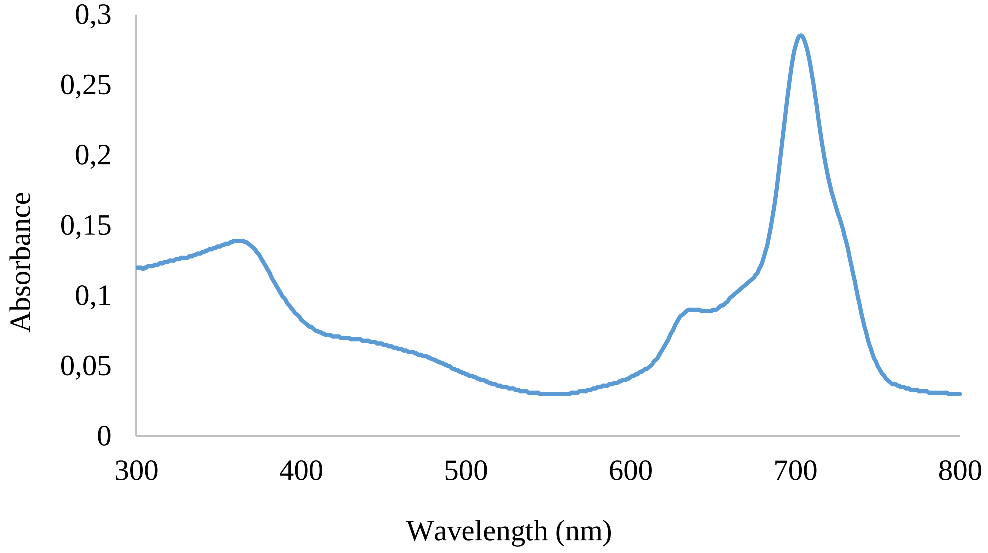

Figure S19 UV–Vis spectra of **AB$_3$TPP-Pc** in DMSO.

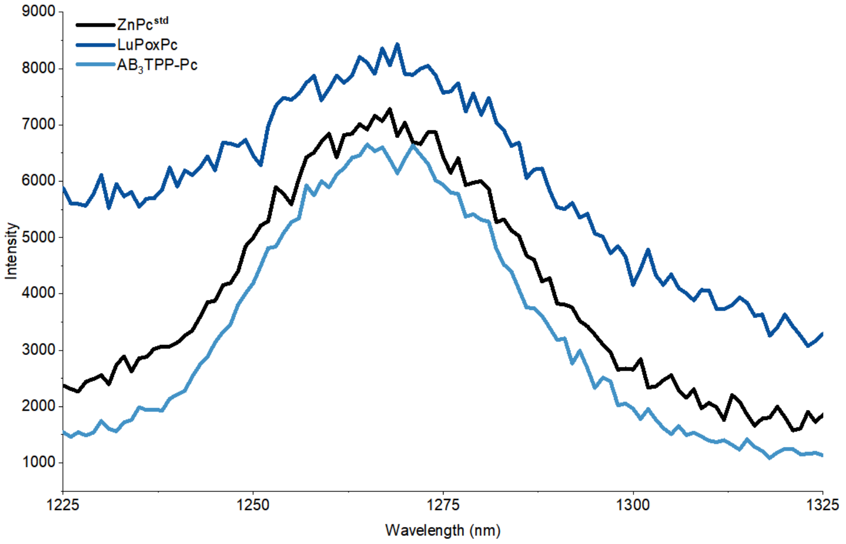

Figure S20. Singlet Oxygen Phosphorescence with sensitization from phthalocyanine derivatives in THF at equal absorbances (0.23) at the wavelength of the maximum of their respective absorbances. ($\lambda_{max,}$/nm; 666, 705, 694 for **ZnPc, LuPcPox(OAc)**, **AB$_3$TPP-Pc**, respectively)

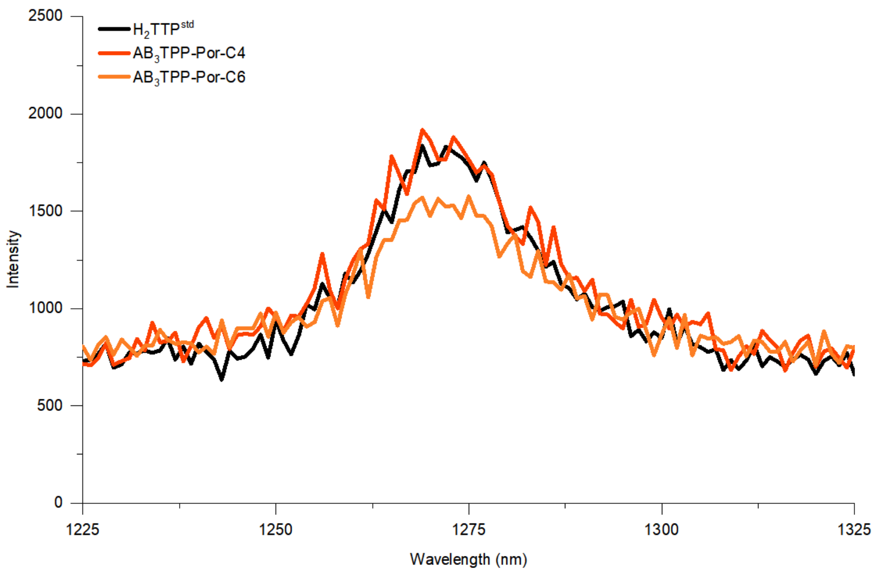

Figure S21. Singlet Oxygen Phosphorescence with sensitization from porphyrin derivatives in THF at equal absorbances (0.23) at the wavelength of the maximum of their respective absorbances. ($\lambda_{max,}$/nm; 415, 420, 420 for **AB_3TPP-Por-C4 and AB_3TPP-Por-C6**, respectively)

References

(1) Williams, A. T. R.; Winfield, S. A.; Miller, J. N. Relative Fluorescence Quantum Yields Using a Computer-Controlled Luminescence Spectrometer. *Analyst* **1983**, *108* (1290), 1067–1071. https://doi.org/10.1039/AN9830801067.

(2) Saka, E. T.; Durmuş, M.; Kantekin, H. Solvent and Central Metal Effects on the Photophysical and Photochemical Properties of 4-Benzyloxybenzoxy Substituted Phthalocyanines. *J. Organomet. Chem.* **2011**, *696* (4), 913–924. https://doi.org/https://doi.org/10.1016/j.jorganchem.2010.10.024.

(3) Ogunsipe, A.; Nyokong, T. Photophysical and Photochemical Studies of Sulphonated Non-Transition Metal Phthalocyanines in Aqueous and Non-Aqueous Media. *J. Photochem. Photobiol. A Chem.* **2005**, *173* (2), 211–220. https://doi.org/https://doi.org/10.1016/j.jphotochem.2005.03.001.

(4) Kaestner, L.; Cesson, M.; Kassab, K.; Christensen, T.; Edminson, P. D.; Cook, M. J.; Chambrier, I.; Jori, G. Zinc Octa-n-Alkyl Phthalocyanines in Photodynamic Therapy: Photophysical Properties, Accumulation and Apoptosis in Cell Cultures, Studies in Erythrocytes and Topical Application to Balb/c Mice Skin. *Photochem. Photobiol. Sci.* **2003**, *2* (6), 660–667. https://doi.org/10.1039/B211348A.
